# Rapid conversion of isoprene photooxidation products in terrestrial plants

**DOI:** 10.1038/s43247-020-00041-2

**Published:** 2020-11-04

**Authors:** Eva Canaval, Dylan B. Millet, Ina Zimmer, Tetyana Nosenko, Elisabeth Georgii, Eva Maria Partoll, Lukas Fischer, Hariprasad D. Alwe, Markku Kulmala, Thomas Karl, Jörg-Peter Schnitzler, Armin Hansel

**Affiliations:** 1 Department of Ion Physics and Applied Physics, University of Innsbruck, Technikerstrasse 25, 6020 Innsbruck, Austria; 2 Department of Soil, Water and Climate, University of Minnesota, 439 Borlaug Hall, St. Paul, MN, USA; 3 Research Unit Environmental Simulation (EUS), Institute of Biochemical Plant Pathology, Helmholtz Zentrum München, Ingolstädter Landstrasse 1, 85764 Neuherberg, Germany; 4 Institute of Biochemical Plant Pathology, Helmholtz Zentrum München, Ingolstädter Landstrasse 1, 85764 Neuherberg, Germany; 5 Institute for Atmospheric and Earth System Research (INAR)/Physics, University of Helsinki, Gustaf Hällströmin katu 2, 00014 Helsinki, Finland; 6 Department of Atmospheric and Cryospheric Sciences, University of Innsbruck, Innrain 52f, 6020 Innsbruck, Austria

## Abstract

Isoprene is emitted from the biosphere into the atmosphere, and may strengthen the defense mechanisms of plants against oxidative and thermal stress. Once in the atmosphere, isoprene is rapidly oxidized, either to isoprene-hydroxy-hydroperoxides (ISOPOOH) at low levels of nitrogen oxides, or to methyl vinyl ketone (MVK) and methacrolein at high levels. Here we combine uptake rates and deposition velocities that we obtained in laboratory experiments with observations in natural forests to show that 1,2-ISOPOOH deposits rapidly into poplar leaves. There, it is converted first to cytotoxic MVK and then most probably through alkenal/ one oxidoreductase (AOR) to less toxic methyl ethyl ketone (MEK). This detoxification process is potentially significant globally because AOR enzymes are ubiquitous in terrestrial plants. Our simulations with a global chemistry-transport model suggest that around 6.5 Tg yr^−^ of MEK are re-emitted to the atmosphere. This is the single largest MEK source presently known, and recycles 1.5% of the original isoprene flux. Eddy covariance flux measurements of isoprene and MEK over different forest ecosystems confirm that MEK emissions can reach 1–2% those of isoprene. We suggest that detoxification processes in plants are one of the most important sources of oxidized volatile organic compounds in the atmosphere.

Biogenic volatile organic compounds (BVOC) are thought to account for 90% of the total VOC emission into the Earth’s atmosphere^1^. Isoprene (C_5_H_8_) is the dominant BVOC with an estimated annual flux of 350–800 Tg yr^−1 2^. Despite this large flux (mainly from broadleaf tree species) the question of why plants emit isoprene is still not fully understood^3^. Recent hypotheses suggest that isoprene acts as a signaling molecule that, by altering gene expressions, strengthens plant defense mechanisms against oxidative and thermal stress^4^. Once in the atmosphere isoprene reacts rapidly with OH radicals, which account for 90% of its total sink^5^. Under typical daytime conditions isoprene is thus converted to oxidized VOC (OVOCs) within 1–2 h^6^. OH preferentially adds to the terminal carbons of isoprene, forming allyl radicals that in collisions with oxygen react to form isoprene peroxy radicals HO–C_5_H_8_O_2_ (ISOPOO). Subsequent oxidative steps depend critically on the ambient abundance of NO_x_ (NO + NO_2_). At elevated NO_x_, ISOPOO predominantly reacts with NO to form methyl vinyl ketone (MVK, yield 30–45%), methacrolein (MACR, yield 20–30%), and isoprene hydroxy nitrate (IHN, yield 13%) plus formaldehyde (HCHO) as the major products^7^. Under low nitrogen oxide (NO_x_) conditions the main product of ISOPOO reacting with HO_2_ is isoprene hydroxy hydroperoxide (ISOPOOH), with the 1,2-ISOPOOH isomer formed in highest yield^5^. Recently, Nguyen et al.^8^ applied the eddy covariance (EC) technique to quantify the rapid dry deposition of ISOPOOH and other oxidized BVOC to a temperate forest. They determined a daytime mean deposition velocity (*v*_d_) for ISOPOOH + IEPOX of 2.5 cm s^−^. Isoprene epoxydiol (IEPOX) is an isomeric photooxidation product of ISOPOOH + OH. ISOPOOH + IEPOX deposition velocities (*v*_d_) were then compared with a resistance model described elsewhere^9,10^ to evaluate whether ISOPOOH + IEPOX is primarily lost to the leaf surface or taken up by the plants through their stomata. The calculated Henry’s law constant for ISOPOOH + IEPOX suggests a very small residual resistance favoring efficient uptake to any liquid surfaces^8^. However, the *v*_d_ for ISOPOOH + IEPOX measured by Nguyen et al.^8^ was too close to both the upper limit for stomata-controlled resistance and the upper limit for deposition without surface resistance to determine which mechanism is dominant. Thus, while direct EC flux measurements quantify the deposition of chemical species to the canopy, the fate of species at the leaf level—and thus any associated ecological impact—cannot be evaluated in this way. In the present study, we combine uptake rates and deposition velocities for 1,2-ISOPOOH and MVK obtained in laboratory experiments on gray poplars (*Populus* × *canescens*) with field observations using the EC technique in natural forest settings. Furthermore, we quantify the gene expression and enzyme activity of the detoxifying NADPH-dependent enzyme alkenal/one oxidoreductase (AOR) in poplar leaves and demonstrate the worldwide dissemination of AOR genes across terrestrial plants. Finally, we apply chemistry-transport modeling to assess atmospheric implications of our findings (see “Methods” section for details). This enables the first leaf-to-global scale description of biosphere–atmosphere exchange for major isoprene photooxidation products, as summarized in Fig. 1.

## Results

### Enclosure experiments.

**Fig. 2:**
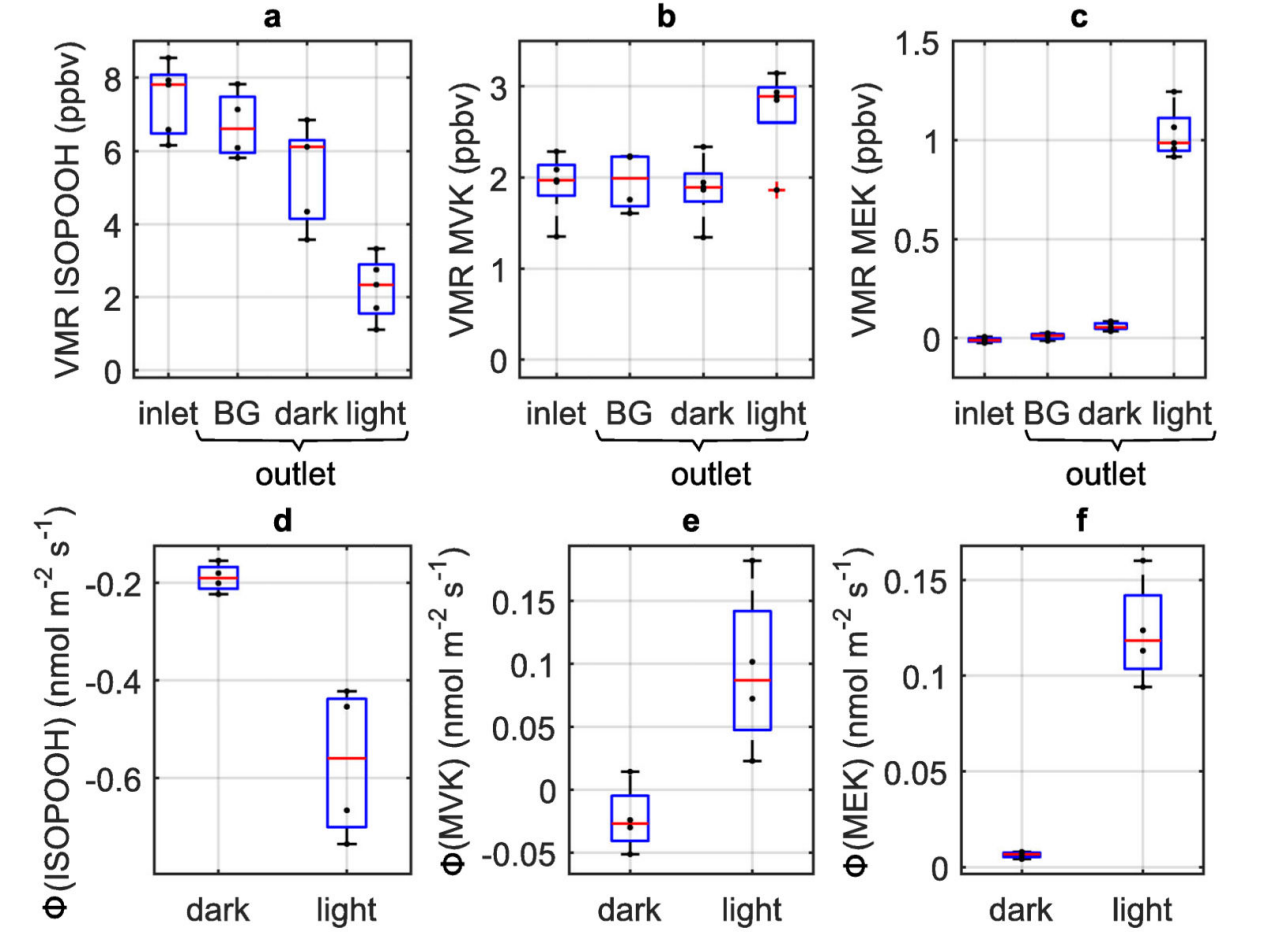
Volume mixing ratios and fluxes of oxidized VOCs (OVOCs) obtained in the poplar fumigation experiments. Box plots of volume mixing ratios (VMR) are shown in panels **a–c** and deposition/emission fluxes (Φ) **d–f** for 1,2-ISOPOOH, MVK, and MEK, respectively. OVOCs were analyzed at the enclosure inlet and subsequently at the enclosure outlet during fumigation of the empty enclosure (BG) and of the darkened/illuminated gray poplar trees. For each box, the red line indicates the median, bars show the minimum/maximum, and the blue box indicates the 25th and 75th percentiles of the sample data (*N* = 5).

In a laboratory study, we investigated the fate of isoprene oxidation products when exposed to poplar leaves (Fig. 2, Supplementary Figs. 1–4). Poplars occur worldwide, both in natural forests and plantations, and are among the most important boreal deciduous tree species. In plant science, poplars represent the most important model system for tree research and were the first tree species for which the genome was described. Many studies, in particular for VOCs, show that the biological and physiological processes studied in poplars can be transferred to other tree species (e.g. refs. ^3,11^). Poplar plants several months in age were selected for the present work because they allow gas exchange analyses and deposition experiments to be carried out in a very well-controlled and reproducible manner. Many VOC emission studies have shown that such results can be transferred to older trees and can hence be used for modeling studies on regional and global scales (e.g. refs. ^2,12^).

The experimental setup is illustrated in Supplementary Fig. 1. Gray poplar plants were placed in an enclosure system and fumigated with synthetic air containing ~450 ppm CO_2_ and ~8 ppbv 1,2-ISOPOOH. Before starting the plant fumigation experiments, we fumigated the empty cuvette with 1,2-ISOPOOH to condition the inner surfaces consisting of Teflon-coated glass. Subsequently, the 1,2-ISOPOOH loss to the surface of the empty enclosure was measured for each individual experiment (see also Supplementary Table 1). The OVOC composition of the inlet and outlet air of the plant enclosure was analyzed with a selective reagent ion time-of-flight mass spectrometer (SRI-TOF-MS). Details of the measurement method (see also Supplementary Table 2), SRI-TOF-MS instrument calibration (see also Supplementary Table 3), and plant material (Supplementary Table 4) can be found in “Methods” section.

The deposition velocity *v*_d,_*_i_* (cm s^−1^) is commonly used to describe trace gas deposition of a substance *i* to vegetation from the atmosphere^13^ and is defined as the ratio between the flux Φ*_i_* (representing the amount of compound *i* deposited to a unit surface area per unit time in μg cm^−2^ s^−1^) and the local concentration *c_i_* (μg cm^−3^).

(1) $$v_{d,i}=-\Phi_{i}/c_{i}$$

Deposition fluxes Φ*_i_* to the plant surfaces inside the enclosure system were calculated from the difference in trace gas mixing ratios between the outlet of the enclosure (*c*_out,_*_i_*) and outlet of the empty enclosure (*c*_out,_*_i_*_,BG_) taking into account the single-sided leaf area (LA) of the enclosed plant and the gas flow (see Supplementary Methods - Enclosure experiment). In this way, we calculated an average 1,2-ISOPOOH deposition velocity of *v*_d_ = 0.12 ± 0.04 cm s^−^ under dark conditions (Fig. 2). The observed daytime value with open stomata (Supplementary Fig. 5) is 0.79 ± 0.25 cm s^−^, close to the 24-h average simulated *v*_d_ of 0.76 cm s^−^ from Nguyen et al.^8^. Almost all deposited 1,2-ISOPOOH thus enters through open stomata into the plant’s inner space, where it will dissolve on wetted surfaces due to its large effective solubility (H* = 1.7 × 10^6^ M atm^−^). Supplementary Fig. 6 shows an experiment with a poplar plant exposed to elevated 1,2-ISOPOOH in the presence of light. Stomatal closure is observed, likely due to stress. Upon stomatal closure, 1,2-ISOPOOH levels in the enclosure increase, indicating that stomatal uptake is most probably the dominant deposition process, and light-induced plant surface uptake is less likely. We further determine from these experiments that 50 ± 15% of deposited 1,2-ISOPOOH is released as the volatile carbonyl MVK (H* = 44 M atm^−^) while the other 50% is converted to MEK (H* = 21 M atm^−^), which is likewise released back into the atmosphere.

Organic hydroperoxides are inherently unstable species. Homolytic cleavage of the weak peroxy bond (O–OH) is a common decomposition mechanism, and can be catalyzed by metals^14^. Recently, we have shown that 1,2-ISOPOOH is efficiently converted to MVK and HCHO on clean stainless steel surfaces^15,16^. We find here from separate laboratory experiments (Supplementary Fig. 7) that a range of different metals catalyze the conversion of 1,2-ISOPOOH to MVK even at room temperature. Chevallier et al.^17^ studied “Fenton-like” reactions of methylhydroperoxides with Fe^2+^ in aqueous solutions. They identified Fe^2+^ + ROOH → Fe^3+^ + RO + OH as the dominant first reaction step. The alkoxy (RO) radicals further rearrange in water solution, and react with dissolved oxygen to form peroxy radicals which decompose, leading to the formation of aldehydes and other products. Assuming that the same reaction mechanism occurs when ISOPOOH dissolves in the liquid phase of the apoplast, low-valence transition metals present in plant cell walls and also dissolved in the apoplast^18^ could catalyze via Fenton-type reactions the conversion of 1,2-ISOPOOH to MVK + HCHO + HO_2_ in plant leaves. Since MVK (in contrast to formaldehyde) has relatively low solubility, it would then be released into the atmosphere through open stomata as its concentration builds up in the liquid phase of the apoplast. In biological systems peroxide functional groups (ROOH) serve as reactive oxygen intermediates that cause oxidative damage and cell death^19^. For example, Polle and Junkermann^20^ found hydroxymethyl hydroperoxide (HMHP) to inhibit peroxidase activity in plant apoplast. Plants exposed to harmful compounds in this way typically release stress-induced volatiles such as methyl salicylate (MeSA). MeSA is an airborne cue inducing pathogen resistance within and between plants^21^, while also enhancing indirect plant defenses against herbivores by attracting their natural enemies^22^. Furthermore, unstressed poplar varieties in general are weak sesquiterpene (SQT) emitters^23^, but SQT emissions are known to increase under various type of abiotic and biotic stresses^24,25^. Here we observe a significant increase in emissions of both MeSA and SQT during 1,2-ISOPOOH fumigation (Supplementary Fig. 8), but only under daylight conditions when stomata are open. Our laboratory experiments therefore indicate that stomatal uptake of 1,2-ISOPOOH causes severe stress to poplar plants and triggers self-defense mechanisms to mitigate oxidative damage. The results also indicate that upon deposition to plants, 1,2-ISOPOOH is converted as a first step to MVK. The exact reaction mechanism of this first step is not known. However, we speculate that the Fenton-type reactions discussed above involving transition metals present in plant cell walls and dissolved in the apoplast^18^ catalyze the conversion of 1,2-ISOPOOH to MVK. As an α,β-unsaturated carbonyl and reactive electrophilic species, MVK itself is toxic to plant tissue due to its high reactivity and ability to form Michael adducts with the thiol and amino groups of biomolecules^26,27^. Previous MVK and MACR deposition experiments using poplars, three different *Quercus* species and houseplants have found both compounds to be immediately lost upon entry through open stomata^28–30^. Tani et al.^29^ observed that the uptake rate correlates strongly with the stomatal conductance. Further, Cappellin et al.^31,32^ found that red oak (a strong isoprene emitter) converts MVK to MEK. Here we find based on additional MVK fumigation experiments with gray poplar plants (Supplementary Fig. 4) that upon stomatal opening MVK fumigation leads to a significant increase in stress-related MeSA and SQT emissions (Supplementary Fig. 8). Furthermore, the MVK detoxification mechanism is highly efficient: all (100 ± 5%) deposited MVK is released as MEK into the atmosphere (Supplementary Fig. 4). As shown by Tani et al.^29,33^ and Cappellin et al.^32^. MEK undergoes leaf uptake with significant lower rates than MVK. Average deposition velocities for MVK of *v*_d_ = 0.044 ± 0.045 and 0.22 ± 0.17 cm s^−^ during dark and light conditions, respectively, are derived here, demonstrating the importance of stomatal fluxes for this process. Poplar fumigation experiments with the 4,3-ISOPOOH isomer did not result in any MEK production. This finding is expected and supports the above proposed mechanism because 4,3-ISOPOOH is converted on metal surfaces to MACR rather than MVK^15,16^.

### Enzymatic reactions within plant tissues.

**Fig. 3:**
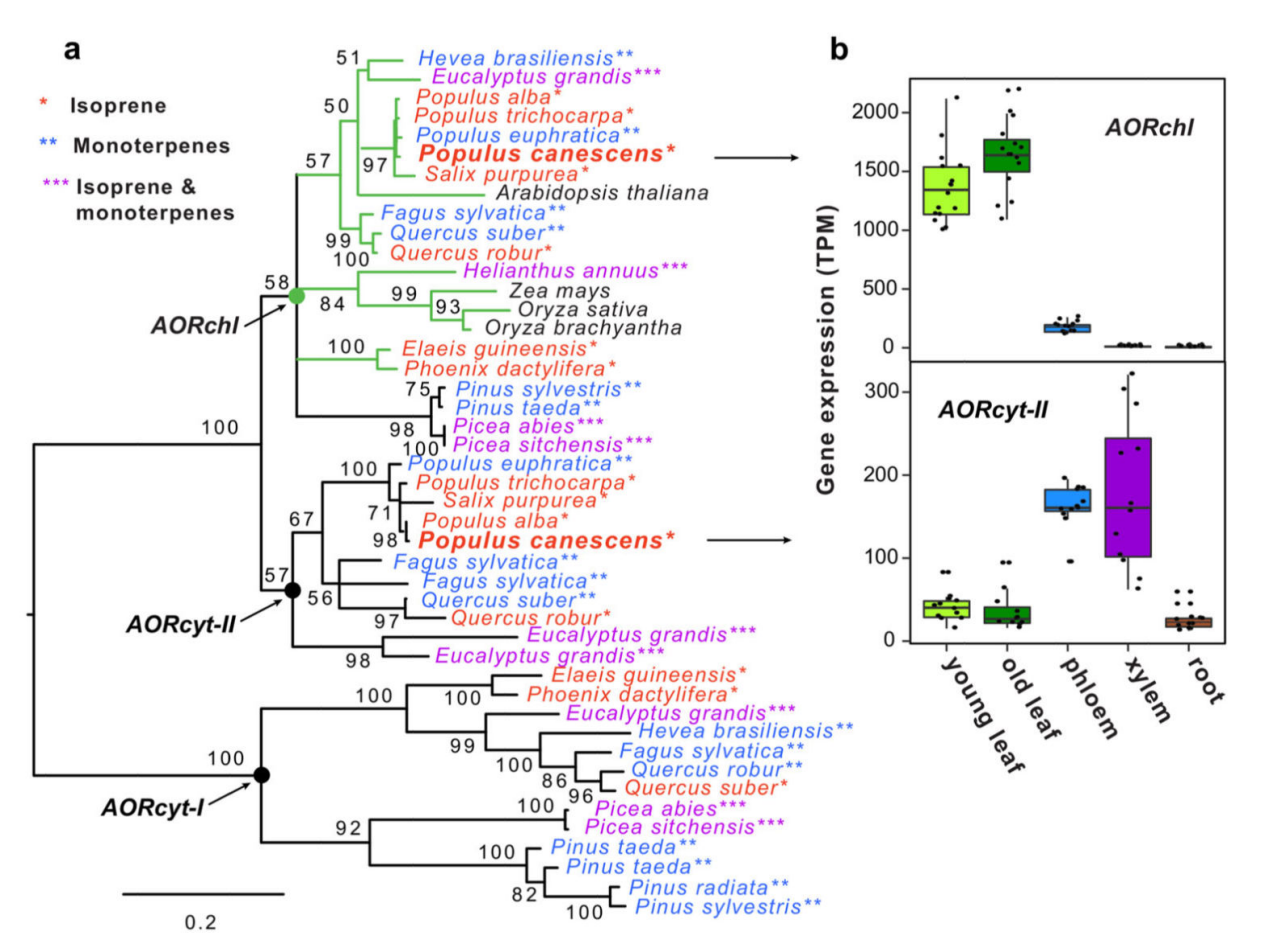
Distribution of genes encoding AOR proteins. The maximum likelihood (ML) phylogenetic tree **a** shows the occurrence of *AORchl, AORcyt-I,* and *AORcyt-II* genes in dominant plant species. Asterisks and coloring of the branch labels indicate species emitting predominantly isoprene, monoterpenes, or both. Numbers at tree branches indicate node bootstrap support. Green branch coloring indicates the presence of plastid-targeting peptides (TP) in the corresponding AOR sequences. Basal nodes of the *AORchl, AORcyt-I,* and *AORcyt-II* ortholog clusters are labeled with solid circles. The scale bar below the tree shows branch length. Box plots **b** show tissue-specific expression of *AORchl* and *AORcyt-II* genes in gray poplar (*Populus* × *canescens*). Error bars show the minimum/maximum.

Several studies have shown that enzymatic reduction of α,β-unsaturated ketones takes place in plant suspension cells and cytosolic fractions of yeast^34,35^. In particular, Yamauchi et al.^36^ identified an NADPH-dependent acrolein-reducing enzyme in cucumber leaves that catalyzes an AOR reaction of the α,β-unsaturated bond. We characterized the in vitro kinetic properties of the poplar AOR (EC 1.3.1.74) enzyme to which we attribute the in planta conversion of MVK to MEK. We obtain Michaelis–Menten constants (substrate concentration at half-maximal enzyme velocity) of 0.049 and 14.15 mM for NADPH and MVK, respectively, and a temperature optimum at 35 °C (Supplementary Fig. 9). This biochemical capability to reduce MVK to MEK is globally present in terrestrial plants, as a phylogenetic survey of genes coding for NADPH-dependent alkenal/on-oxidoreductases (AOR) demonstrates (Fig. 3, Supplementary Fig. 10, Supplementary Table 5). One gene encoding AOR is localized in the chloroplast (AORchl), and two more are located in the cytosol (AORcyt-I and AORcyt-II; Fig. 3b and Supplementary Fig. 10). The distribution of AORchl and AORcyt in plants is independent of isoprene and monoterpene emissions (Fig. 3a): AOR genes are ubiquitous in all land plants including mosses, clubmosses, gymnosperm, and flowering plants (Supplementary Fig. 10). Our gene expression analyses for gray poplar show that AORchl is predominantly expressed in leaf tissue, while AORcyt-II is expressed in the stem (phloem and xylem) (Fig. 3b). Under normal environmental conditions^37^ the total gene expression rates of AORchl are more than six times those of AORcyt-II. We analyzed in situ AOR activity in leaf extracts from poplar plants fumigated with either 1,2-ISOPOOH or MVK (Supplementary Fig. 11). The fact that we do not see any change to the in situ AOR activity as a result of exposure to 1,2-ISOPOOH and MVK (Supplementary Fig. 11) may be due to the fact that our observation period was too short and missed a stress-induced response. However, AOR also belongs to a group of proteins that are post-translationally modified (PTM) by S-nitrosylation at certain cysteine residues under stress (Supplementary Fig. 12)^38^, which may change in vitro enzyme activity. In planta, i.e. the intact leaves as we used for the gas exchange analyses, the AOR activity is variable depending on actual leaf temperature, light energy providing electrons in the form of NADPH for the reduction of MVK to MEK, and the presence of MVK, which is provided by deposition or degradation of 1,2-ISOPOOH. Overall, the phylogenetic analysis and the measurements of AOR activity in our model tree system imply that green biomass across the globe is able to convert MVK to MEK.

### Impact on regional and global budgets of OVOCs.

**Fig. 4:**
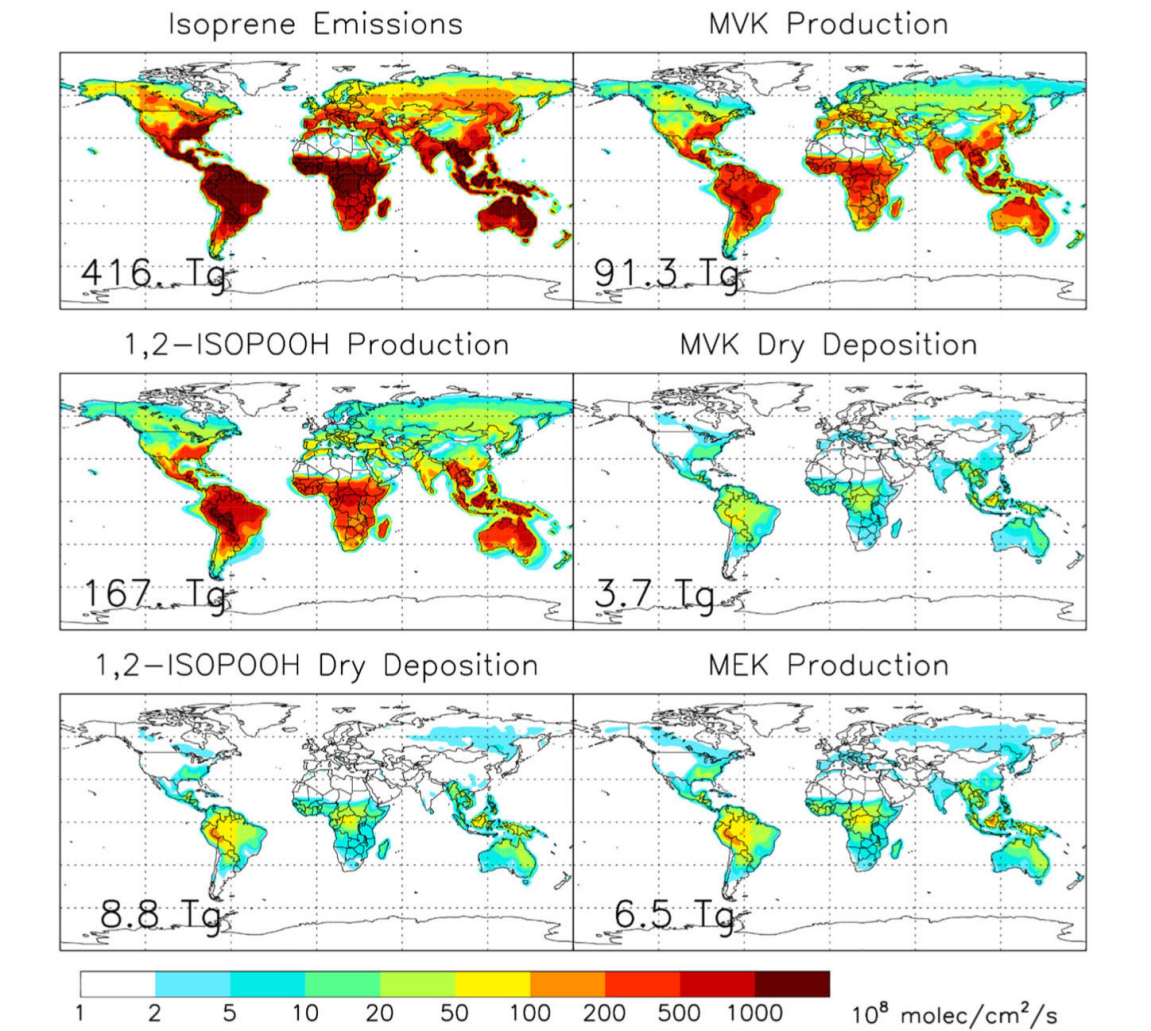
Simulated global isoprene emissions along with the production and deposition of key oxidation products (1,2-ISOPOOH, MVK). Simulations were performed by the GEOS-Chem CTM for 2017 (see “Methods” section for model details). Reconciling the model deposition velocities using our experimentally-obtained *v*_d_, and assuming that 100% of dry deposited MVK and 50% of dry deposited 1,2-ISOPOOH undergoes conversion in plants, reveals a global MEK source of 6.5 Tg yr^−1^ (and recycling 1.5% of the isoprene flux).

We performed global simulations using the GEOS-Chem chemical transport model (CTM) (v12.1.1, https://doi.org/10.5281/zenodo.2249246) to assess how dry deposition of 1,2-ISOPOOH and MVK (as the main isoprene oxidation products) impact the atmospheric MEK budget (see “Methods” section for model details). A global emission of 416 Tg isoprene is simulated by the model for 2017, leading to the photochemical production of 91.3 Tg MVK and 167 Tg 1,2-ISOPOOH (Fig. 4). By default, GEOS-Chem uses a modified resistance-based approach from Wesely^9^ to calculate dry deposition velocities. The performance of this approach depends on knowledge about atmospheric stability, surface conditions, and the solubility and reactivity factors *f*_0_ for compounds of interest. The stomatal fraction of 1,2-ISOPOOH dry deposition thus simulated by the model generally ranges from <5% to 30% over land (Supplementary Fig. 13), which does not agree with our findings here (*v*_d_ = 0.79 cm s^−^ during daytime versus only 0.12 cm s^−^ in the dark). We therefore question how accurately the default model scheme is able to separate stomatal versus non-stomatal deposition for OVOCs. Instead, we prescribe the modeled 1,2-ISOPOOH and MVK deposition velocities over land based on the results from our laboratory measurements employing constant daytime (nighttime) deposition velocities of 0.22 (0.044) cm s^−^ for MVK and 0.79 (0.12) cm s^−^ for 1,2-ISOPOOH. Over oceans the default model scheme is used. Since the laboratory chambers were well-mixed, this corresponds to an assumption that canopy uptake for these compounds is controlled by surface and molecular diffusion resistance, an assumption strongly supported by prior work^8^. Figure 4 shows the resulting simulated global deposition of MVK (3.7 Tg yr^−^) and 1,2-ISO-POOH (8.8 Tg yr^−^). Assuming based on our measurements that 100% of dry deposited MVK and 50% of dry deposited 1,2-ISOPOOH undergoes conversion to MEK, the model yields a global MEK flux of 6.5 Tg yr^−^ (1.5% of isoprene emissions, Supplementary Fig. 14), making this mechanism the largest known MEK source to the atmosphere^39^. In turn, a fraction of that MEK will return to the ecosystem via further dry deposition (e.g. refs. ^29,32,33^).

For comparison, we performed a GEOS-Chem base-case run (Supplementary Fig. 15) in which the default Wesely scheme was used to simulate 1,2-ISOPOOH and MVK dry deposition. Assuming that only the stomatal component of deposition leads to MEK then yields a global source of 3 Tg yr^−^. We view this as a lower limit given the apparent underestimate of OVOC stomatal uptake in the default model. On the other hand, assuming that all dry deposited MVK and 50% of all dry deposited 1,2-ISOPOOH undergoes conversion results in an MEK source of 14 Tg yr^−^ (upper limit).

### Eddy covariant (EC) VOC flux measurements.

To evaluate these findings, we performed EC flux measurements of isoprene and MEK at two sites. The first is the SMEAR II station in Hyytiälä (Finland) (Supplementary Fig. 16), which is surrounded by low isoprene-emitting plants (mainly Scots pine *Pinus sylvestris*) with an average daytime isoprene flux of 1 nmol m^−2^ s^−1^ in spring. Details of the eddy covariant VOC flux measurements can be found in “Methods” section and Supplementary Methods - Eddy covariance VOC flux measurements. The second EC flux measurements were performed at PROPHET (US) (Supplementary Fig. 17), which is a high isoprene emission site in a mixed deciduous/coniferous forest. Typical daytime isoprene flux values at PROPHET reach ~10 nmol m^−2^ s^−1^ (Supplementary Fig. 17). Flux data for isoprene and MEK were obtained using the EC method, correlating fast mixing ratio changes (~10 Hz) with vertical wind velocities. The high sensitivity achieved by the PTR3-TOF for MEK (>12,000 cps/ppbv) enabled the first accurate measurement of these fluctuations at the low isoprene-emission site. In general, the recent development of very sensitive, fast, and quantitative mass spectrometry-based detectors such as PTR-QiTOF and PTR3-TOF^40^ was essential for EC measurements of MEK. We find that MEK emissions are 1.8% and 0.9% those of isoprene, at these sites, respectively. This is in excellent agreement with the model best-estimate (1.5%; see also Supplementary Fig. 14) and supports the global importance of this mechanism for plant oxidative protection and for the biogenic MEK atmospheric source.

## Discussion

Ketones are an important class of OVOC with sufficiently long atmospheric lifetimes to be transported to the upper troposphere. MEK, like acetone, photolyzes in the near UV region; as a result, these ketones provide an important source of HO_x_ (OH + HO_2_) radicals in the dry upper troposphere^41,42^. Degradation of MEK in the lower atmosphere generates toxic and photo-active compounds such as acetaldehyde, a peroxyacetylnitrate (PAN) precursor, and formaldehyde^43^. Global sources of MEK, the second most abundant atmospheric ketone, are still not fully understood. Singh et al.^39^ presented a first assessment of MEK sources and sinks based on aircraft measurements over the Pacific Ocean. Based on their derived MEK atmospheric burden and an assumed 7-day lifetime due to reaction with OH and photolysis, they inferred a total global source of 11 Tg yr^−1^. They further estimated MEK sources of 1–3 Tg yr^−1^ each from hydrocarbon oxidation and biomass burning, and of 0–1 Tg yr^−1^ from oceanic emissions. The latter estimate is supported by more recent analysis by Brewer et al.^44^. A residual unexplained source of 7 Tg yr^−1^ was then attributed by Singh et al.^39^ to direct biogenic emissions. Our work combining dedicated laboratory, field, and model analyses derives a global biogenic MEK source of 6.5 Tg yr^−1^, and provides a new biochemical and genetic basis for understanding the processes driving plant–atmosphere exchange of 1,2-ISOPOOH, MVK and MEK, and for modeling this exchange at regional and global scales.

Plants possess the ability to absorb OVOCs from the atmosphere and actively convert toxic chemicals to less toxic species, which can then be re-emitted into the atmosphere. Here we show that following uptake the toxic isoprene oxidation products 1,2-ISOPOOH and MVK are converted and re-emitted as MEK. The genes coding for the responsible enzyme alkenal/an oxidoreductase (AOR) are ubiquitously distributed in the plant kingdom. For MEK this process leads to an atmospheric input of about 6.5 Tg, the single largest known term in the global MEK budget and re-emitting 1.5% of the original isoprene emissions. Our findings thus support and provide a mechanistic underpinning to understand the biogenic emissions inferred by Singh et al.^39^, and implicate detoxification as the most important source process in the global MEK budget. The global MEK estimate has uncertainties due to the underlying up-scaling errors. These uncertainties include those linked with the enclosure measurements as well as AOR activity changes with temperature. We found in enclosure measurements that stomatal uptake is the dominant route, but leaf surface conversion on wet surfaces may also occur under real world conditions. However, for many years scientists have used plant enclosure experiments in combination with field-based flux measurements to better asses global BVOC emissions. For example, Guenther et al.^45^ compared BVOC flux estimates from enclosure and ambient measurements and found agreement to within a factor of two. These findings suggest a significant modification of OVOCs at the biosphere–atmosphere interface. Traditional dry deposition schemes that are widely used in atmospheric chemistry models do not capture the uptake process, and new mechanistic approaches should be developed to capture the complex behavior of bi-directional exchange at the biosphere–atmosphere interface.

## Methods

### SRI-TOF-MS.

The SRI-TOF-MS is a custom-built instrument that represents an advanced version of the PTR-TOF-MS system described by Graus et al.^46^, and features the capability to switch between different reagent ions within seconds. Additionally, we replaced the standard metal drift rings of the reaction chamber with chemically inert conductive PEEK rings. This feature and the high flow rate through the reaction chamber (~800 ml min^−1^ instead of 10–30 ml min^−1^ in standard PTR-MS instruments) were essential for minimizing metal-catalyzed decomposition of 1,2-ISOPOOH as observed in standard PTR-MS instruments^15^. The OVOC composition of the inlet and outlet air of the plant enclosure system was sequentially analyzed by SRI-TOF-MS operated with ammonia (NH_4_^+^) and occasionally with nitronium (NO^+^) or hydronium (H_3_O^+^) reagent cations. The SRI-TOF-MS was operated at a constant temperature (35 °C) and pressure (2.3 mbar) in the drift tube. Different drift voltages of 250, 300, and 500 V resulting in *E/N* values of 45 Td (NH_4_^+^-mode), 54 Td (NO^+^-mode), and 90 Td (H_3_O^+^-mode), respectively, were used (*E* is the electric field strength, while *N* is the number gas density; 1 Td = 10^−17^ V cm^2^). Raw data analysis was performed with the PTR-TOF-Data Analyzer^47^ and the data processing routine described in Breitenlechner et al.^40^. For details about chemical ionization mechanism and calibration of the SRI-TOF-MS see the Supplementary Methods—Enclosure experiments and Supplementary Table 4.

### PTR3-TOF

In the recently developed *PTR3-TOF* instrument^40^ a discharge ion source is coupled to a contact-free inlet system through the novel reaction chamber at 80 mbar. The inlet system is operated at high sample gas flow rates. The PTR3 front portion is coupled to a TOF from TOFWERK mass analyzer. The instrument has sensitivities of up to 20,000 cps/ppbv and a mass resolution of ~8000 m/Δm. VOCs were ionized via reactions with H_3_O^+^(H_2_O)*_n_* primary ions. Flux data for isoprene and MEK were obtained using the EC method, correlating fast mixing ratio changes (~10 Hz) with vertical wind velocities. The high sensitivity achieved by the PTR3-TOF for MEK (>12,000 cps/ppbv) enables more accurate measurement of these fluctuations than was previously possible. Calibration details are described in the Supplementary Methods—Eddy covariance VOC flux measurements.

### Plant material.

In the present study we used 4-month-old gray poplars (*Populus* × *canescens* INRA clone 7171-B4; syn. *P. tremula* × *P. alba* (Aiton.) Smith). Gray poplar shoots had been amplified by micropropagation on half-concentrated MS medium and further cultivated at the Helmholtz Zentrum München to an age of 3 months as described elsewhere^48^. Before being used in the experiments, the poplar plants were grown in a greenhouse at the Institute of Microbiology of the University of Innsbruck for 1–4 weeks under natural light conditions. One day prior to the experiment, the plants were transferred to the laboratory to adapt to the light conditions (12-h photoperiod with an approximate PAR of 400 ¼mol photons m^−2^ s^−1^). All plants were well watered and showed no visible illness symptoms.

### Determination AOR activity.

To determine apparent in vitro AOR activities in poplar leaf protein extracts, we deep-froze three mature leaves (leaf # 9–11, below the apex) from each poplar plant with liquid nitrogen at the following experimental phases: (1) before the experiments, (2) immediately after the experiment, i.e., after 24 h of ISOPOOH fumigation (12 h illuminated and 12 h in the dark), and (3) 24 h after the end of the enclosure experiment. The apparent in vitro alkenal/one oxidoreductase (AOR) activity was assessed as described in Yamauchi et al.^49^. Frozen leaf material was ground in liquid nitrogen using a dismembrator ball mill (B. Braun Biotech International, Melsungen, Germany). 200 mg of leaf powder were extracted in 4 ml of plant extraction buffer (100 mM Tris/HCl, pH 8.0, 20 mM MgCl_2_, 100 mM CaCl_2_, 1.5% PEG1500 (w/v), 5% (v/v) glycerol, 0.1% (v/v) Tween-20, and 20 mM DTT) with 200 mg polyvinylpolypyrrolidone (PVPP), stirred for 15 min on ice (4 °C) and centrifuged for 15 min at 20,000 × *g*. The supernatant was purified on Sephadex G-25 PD-10 columns (GE Healthcare, Solingen, Germany) equilibrated with enzyme buffer (50 mM Tris/HCl, 20 mM MgCl_2_, 5% (v/v) glycerol, 2 mM DTT)^50^. Protein concentrations were determined by the Bradford assay using a Roti-Quant Kit (Carl Roth, Karlsruhe, Germany). Kinetic properties of AOR were initially characterized with changing concentrations of NADPH and MVK and at changing assay temperatures (Supplementary Fig. 9). AOR activities were finally measured in crude protein extracts in the presence of 0.15 mM NADPH (under a saturating NADPH concentration; 3-times Michaelis–Menten constant (0.049 mM)), monitoring changes at A340 nm with a substrate concentration of 30 mM MVK (dissolved in enzyme buffer at a saturating MVK concentration of 2-times Michaelis–Menten constant (14.15 mM)) against a control without MVK according to Yamauchi et al.^49^ in a final assay volume of 1 mL. From extracts of 7 (1,2-ISOPOOH fumigation) and 5 (MVK fumigation) biological replicates three technical replicates were measured. A description of the construction of the AOR phylogenetic tree and AOR gene expression analysis can be found in the Supplementary Methods—Construction of AOR phylogenetic trees and AOR gene expression analysis.

### EC flux measurements

The first *EC flux measurement* site is situated at the SMEAR II station in Hyytiälä, Finland. Vegetation around the site is dominated by Scots pine (*P. sylvestris*) with a canopy height of ~15 m. The ground level vegetation consists of lingonberry (*Vaccinium vitis-idaea*), blueberry (*V. myrtillus*), and mosses (*Pleurozium scheberi, Dicramum polysetum*)^51^. Flux data for isoprene and MEK were obtained with the PTR3 using the eddy covariance (EC) method during spring 2016. The PTR3 sampled air through a specially designed 5 m long tube with a high flow rate on top of a 35 m tower. Wind speed measurements were taken 0.5 m above the inlet opening by a METEK USA-1 sonic anemometer at 20 Hz. Wind direction and speed were cross checked against the SMEAR II station instrumentation.

The second EC flux measurement site is situated at the University of Michigan Biological Station (UMBS). Isoprene, MEK, and other VOCs were measured by PTR-QiTOF as part of the PROPHET-AMOS field study in July 2016^52,53^. The 34 m PROPHET tower (45.559°N, 84.715°W, 232 m elevation) is located in a mixed deciduous/coniferous forest (canopy height ~23 m) with an upper canopy dominated by aspen, birch, and red oak, and a lower canopy consisting mainly of white pine, red maple, beech, and red oak^54,55^. Net above-canopy VOC fluxes were measured each hour by eddy covariance throughout the campaign. Sampling, instrument operation, calibration procedures, data processing, and QA/QC are described in detail elsewhere^52^.

### GEOS-Chem model.

*GEOS-Chem* (v12.1.1, https://doi.org/10.5281/zenodo.2249246; www.geos-chem.org) is a global 3D CTM driven by assimilated meteorological fields (Goddard Earth Observation System Forward Processing product; GEOS-FP) from the NASA Global Modeling and Assimilation Office (GMAO). The GEOS-FP data have native horizontal resolution of 0.25° latitude × 0.3125° longitude with 72 vertical layers. For the year 2017 global simulations presented here, we degrade the horizontal resolution to 2° × 2.5° and use a 15-min transport time step. We use the TPCORE advection algorithm^56^, convective mass fluxes from the GEOS-FP archive^57^, and the non-local boundary layer mixing scheme described by Lin and McElroy^58^.

Wet deposition in GEOS-Chem is as described by Amos et al.^59^, and includes convective scavenging as well as rainout and washout by large-scale precipitation. The default dry deposition in GEOS-Chem employs the resistance-in-series scheme formulated by Wesely^9^ and subsequently updated and implemented by Wang et al.^60^. Deposition velocities are computed in the model as a function of aerodynamic, boundary layer and surface resistances, with the first two computed from relevant molecular and meteorological parameters as detailed previously^60^. Surface resistances incorporate stomatal, cuticular, lower canopy, and ground surface pathways, and are likewise derived according to local environmental conditions^60,61^. Relevant parameters for 1,2-ISOPOOH and MVK include H* values of 1.7 × 10^6^ and 44 M/atm, respectively, and reactivity (*f*_0_) values of 1.0 for both species.

The GEOS-Chem chemical mechanism (publicly available online via https://doi.org/10.5281/zenodo.2249246) includes comprehensive HO_x_–NO_x_–VOC–ozone–halogen chemistry coupled to aerosols and incorporates current JPL/IUPAC recommendations with recent updates for isoprene chemistry^62,63^, peroxyacetyl nitrate^64^, and Criegee chemistry^65^. Photolysis frequencies are calculated for 18 wavelength bins spanning 177–850 nm using the Fast-JX algorithms developed by Bian and Prather^66,67^. Biogenic VOC emissions are computed online based on the Model of Emissions of Gases and Aerosols from Nature (MEGANv2.1)^2^ as described by Hu et al.^68^. Specifically, emissions for each model grid cell are computed from vegetation-specific emission factors and multiplicative non-dimensional activity factors accounting for emission dependencies on light, temperature, LA, and leaf age^2^. Biogenic MEK fluxes are computed separately by applying our laboratory measured-yields (100% and 50%, respectively) to the modeled MVK and ISOPOOH deposition fluxes as detailed in the main text. Global anthropogenic emissions of VOCs, CO, NH_3_, NO_x_, SO_2_, and aerosols are from the Community Emissions Data System (CEDS) inventory^69^ overwritten by regionally specific inventories for North America^70^, Asia^71^, and Africa^72^. Biogenic soil NO_x_ emissions are from Hudman et al.^73^.

## Supplementary Material

SI

## Figures and Tables

**Fig. 1:**
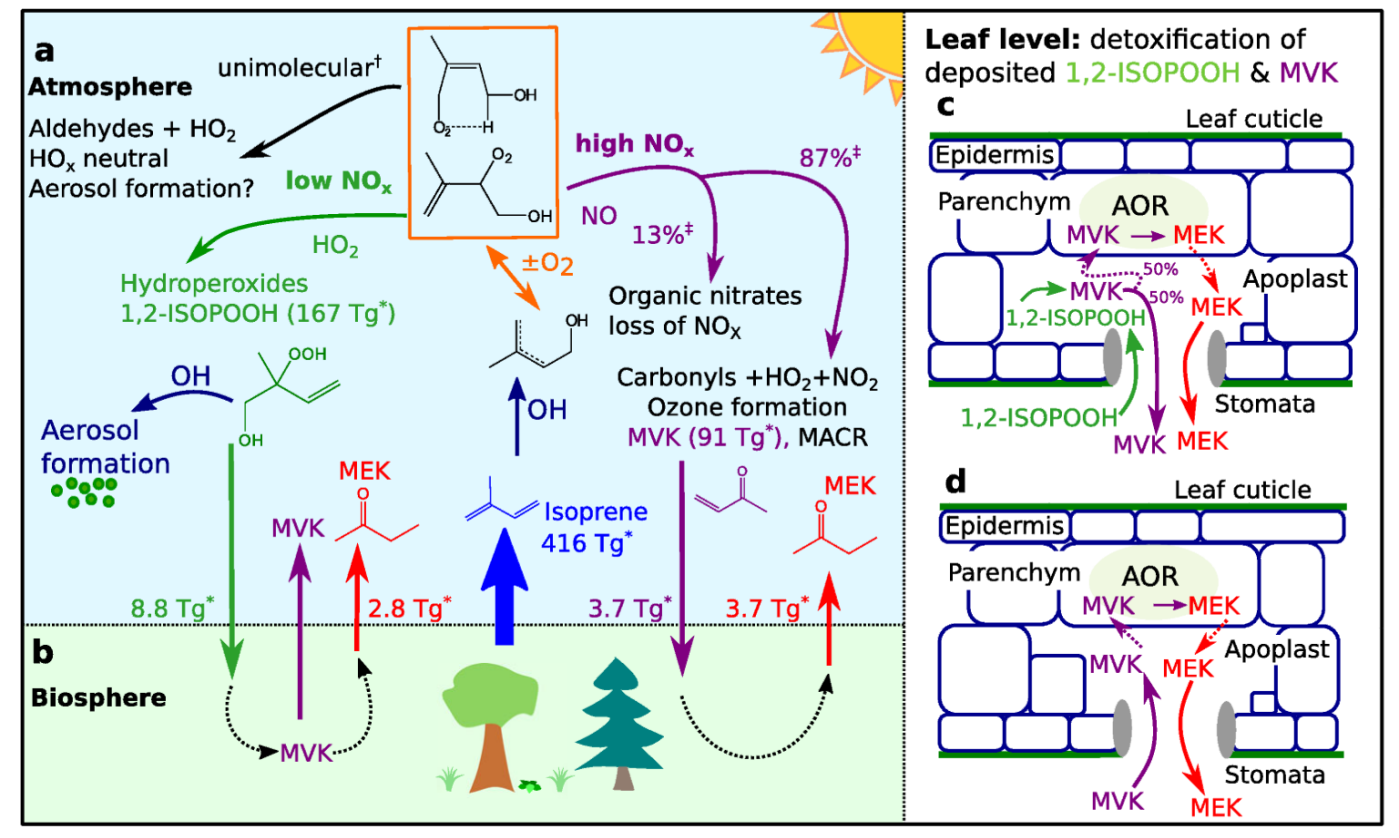
Organic carbon exchange of major isoprene photooxidation products between the biosphere and the atmosphere on a global scale. Under low nitrogen oxide (NO_x_) conditions 1,2-ISOPOOH is preferentially formed, producing epoxides that then react with OH and contribute to aerosol formation^74^. At high NO_x_ the production of carbonyls such as methyl vinyl ketone (MVK) supports ozone formation^5^ (panel **a**). Dry-deposited 1,2-ISOPOOH and MVK (panel **b**) is instantaneously detoxified within the plant leaf (panels **c, d**) via the enzyme alkenal/one oxidoreductase (AOR). EC measurements in natural forest settings confirm our modified GEOS-Chem model results (indicated with *) that ~6.5 Tg methyl ethyl ketone (MEK) (corresponding to 1.5% of the isoprene source) is emitted into the atmosphere in this way. This is the single largest known MEK source on a global scale. ^†^Values from ref. ^75^. ^‡^Values from ref. ^7^.

## Data Availability

The data that support the findings of this study are openly available in Zenodo (https://doi.org/10.5281/zenodo.4010848).

## Code availability

We performed global simulations using the GEOS-Chem chemical transport model (CTM) (v12.1.1, https://doi.org/10.5281/zenodo.2249246), which is freely available at www.geos-chem.org.
