# Supplementary material for: Rapid conversion of isoprene photooxidation products in terrestrial plants
Source: Commun Earth Environ. Author manuscript; Available in PMC 2021 Feb 19. (PMC7894407; doi:10.1038/s43247-020-00041-2)
Supplement: SI [file NIHMS1669554-supplement-SI.pdf]

Supplementary Information for

Rapid conversion of isoprene photooxidation products in terrestrial plants

Authors: Eva Canaval, Dylan B. Millet, Ina Zimmer, Tetyana Nosenko, Elisabeth Georgii, Eva Maria Partoll, Lukas Fischer, Hariprasad D. Alwe, Markku Kulmala, Thomas Karl, Jörg-Peter Schnitzler, Armin Hansel*

Correspondence to: armin.hansel@uibk.ac.at

This PDF file includes:

Supplementary Methods
Supplementary Figures 1 to 17
Supplementary Tables 1 to 5
Supplementary References

Table of Contents

Supplementary Methods – Enclosure Experiment 3
Experimental Design.....3
Characterization of 1,2-ISOPOOH deposition to the empty enclosure.....3
Photolytic loss of 1,2-ISOPOOH.....4
Four-step protocol for the 1,2-ISOPOOH fumigation experiment.....4
Representative poplar fumigation experiment.....5
Calculation of emission dynamics.....5
Liquid Calibration Unit (LCU).....6
Infrared gas analyzer (IRGA).....6
Chemical ionization utilizing SRI-ToF-MS.....6
Calibration of the NH_4^+-mode for SRI-ToF-MS.....7
Impurity determination for fumigation experiments.....7
Supplementary Methods - Construction of AOR Phylogenetic Trees and AOR Gene Expression Analysis.....7
Supplementary Methods - Eddy Covariance VOC Flux Measurements.....8
Calibration and data analysis of the PTR3.....8
Statistical analysis.....8
Supplementary Figure 1. Experimental design of enclosure measurements.....9
Supplementary Figure 2. Deposition rate to enclosure walls.....10
Supplementary Figure 3. Representative laboratory experimental run.....11
Supplementary Figure 4. Volume mixing ratios (VMR) and fluxes (Φ) of MVK and MEK in MVK fumigation experiments.....12
Supplementary Figure 5. Average net CO_2 assimilation and transpiration rates for 1,2-ISOPOOH fumigated gray poplars.....13
Supplementary Figure 6. Stomatal closing under light conditions during a poplar experiment. 14
Supplementary Figure 7. 1,2-ISOPOOH reduction on different metals.....15
Supplementary Figure 8. MeSA and SQT signals following 1,2-ISOPOOH/MVK fumigation 16
Supplementary Figure 9. Kinetic properties of the gray poplar AOR (EC 1.3.1.74, NADPH-dependent alkenal/one oxidoreductase) activity *in vitro*. 17
Supplementary Figure 10. AOR phylogenetic tree.....18
Supplementary Figure 11. *In vitro* AOR activity in fumigated gray poplar leaves.....19
Supplementary Figure 12. Post-translational modification (PTM) of the *Populus x canescens* AORchl protein.....20
Supplementary Figure 13. Stomatal fraction of 1,2-ISOPOOH deposition.....21
Supplementary Figure 14. MEK yield as simulated by GEOS-Chem.....22
Supplementary Figure 15. GEOS-Chem run with default dry deposition treatment.....23
Supplementary Figure 16. EC flux measurements of isoprene and MEK at low isoprene emission site.....24
Supplementary Figure 17. EC flux measurements of isoprene and MEK at high isoprene emission site.....25
Supplementary Table 1. Wall deposition rates.....26
Supplementary Table 2. Four-step protocol of the ISOPOOH fumigation experiment.....26
Supplementary Table 3. Sensitivities of the SRI-ToF-MS.....26
Supplementary Table 4. Plant details.....27
Supplementary Table 5. Sequence accession and BVOC emission information.....28
Supplementary references.....31

Supplementary Methods – Enclosure Experiment

Experimental Design

The experimental setup is illustrated in Supplementary Figure 1. Gray poplar plants (see Methods - *plant material*) were placed in an enclosure system consisting of a glass desiccator (Schott Duran ©) of 17.3 L volume turned upside-down. The inner surface of the desiccator was coated with Teflon (PFC 801A, Cytonix, USA) in order to minimize surface deposition of oxidized VOCs (OVOCs). The enclosure was placed on two PTFE ground plates equipped with a groove and tongue and ports for air inlet and air sampling, respectively. The plant stem and a Teflon coated K type thermocouple were fed through a central notch in the plates. Possible leaks were sealed with a Teflon tape. A Teflon coated fan (Propeller Stirrer Shaft, 6.5 mm chucking diameter, Bohlender GmbH, Grünsfeld, Germany) connected to a 12 VDC motor was used for turbulent air mixing inside the enclosure. For the entire setup, only chemically inert materials such as PTFE, PFA and PEEK were used. All tubing was light-shielded with pipe insulation to exclude unwanted photolysis of 1,2-ISOPOOH. The plants were fumigated with synthetic air (5.0 grade, Messer Austria GmbH, Gumpoldskirchen, Austria) that was mixed with CO_2 (4.8 grade, Messer Austria GmbH, Gumpoldskirchen, Austria) resulting in a CO_2 volume mixing ratio of on average ~450 ppm. Before entering the enclosure, the air was flushed through a liquid calibration unit (LCU, see below) (Ionicon Analytik, Innsbruck, Austria) to humidify the air and to add a defined quantity of a solution of a synthetic ISOPOOH standard (*1*) in deionized water (2.9 µL ISOPOOH in 100 mL deionized water (v/v)). The ISOPOOH standard consisted entirely of the 1,2-ISOPOOH isomer (kindly provided by the Frank Keutsch group, Harvard University, Boston MA) (*1*). Air composition at the inlet and outlet of the enclosure was alternately analyzed with the SRI-ToF-MS (see below) and an infrared-gas-analyzer (IRGA, see below).

Characterization of 1,2-ISOPOOH deposition to the empty enclosure

In order to quantify possible surface deposition and decomposition of 1,2-ISOPOOH on the Teflon-coated surface of the empty glass enclosure and on Teflon tubing material, we performed 1,2-ISOPOOH fumigation experiments with the empty enclosure. Before starting the plant fumigation experiments, we fumigated the empty cuvette with 1,2-ISOPOOH to condition the inner surfaces. Subsequently, the 1,2-ISOPOOH loss to the surface of the empty enclosure was measured for each individual experiment. The enclosure was flushed with humidified air (RH ~35 %) at room temperature containing 7.8±1.0 ppb 1,2-ISOPOOH. For an estimation of the 1,2-ISOPOOH loss to the empty enclosure, we modelled the deposition rate to the surfaces according to (*2*):

$$k_{dep,surface} = ln\left(\frac{c_{in,ISOPOOH}}{c_{out,ISOPOOH}}\right) \times \frac{1}{\tau}, [s^{-1}] \quad (1)$$

where $c_{in,ISOPOOH}$ [nmol mol^{-1}] is the volume mixing ratio (VMR) of 1,2-ISOPOOH determined in the enclosure inlet air, $c_{out,ISOPOOH}$ [nmol mol^{-1}] is the VMR of 1,2-ISOPOOH measured at the enclosure outlet and τ [s] represents the residence time for a single exchange of the air in the enclosure (see Supplementary Table 1 and Supplementary Figure 2 for an overview). Under well-mixed conditions, which were achieved with the Teflon fan, the residence time τ can be expressed as the ratio of the enclosure volume $V_{enclosure}$ to the enclosure inlet gas flow F (*3*):

$$\tau = \frac{V_{enclosure}}{F}, [min] \quad (2)$$

According to (*4*) it takes 5 τ to exchange 99% of the air in the enclosure. The residence time in our fumigation experiments was on average 4.5 min, thus requiring 22.5 min to reach 99% air exchange.

Supplementary Figure 2 depicts a typical change in 1,2-ISOPOOH deposition rate $k_{dep,surface}$ over time. At the beginning of the experiment, when surfaces are virtually free of 1,2-ISOPOOH, $k_{dep,surface}$ is 0.25 molecules s^{-1}. Subsequently, the deposition rate decreases exponentially over time. 60 min after starting the 1,2-ISOPOOH fumigation the deposition rate becomes smaller than 0.01 molecules s^{-1} and is neglected for further analysis. The characterization of the empty enclosure system revealed a far slower adaption to new experimental conditions for highly water-soluble compounds such as 1,2-ISOPOOH compared to more volatile and less soluble compounds. For example, MVK and MEK are typically adjusted after 23 minutes, which is the time required to completely (> 99 %) exchange the gas in the enclosure.

We used equation (1) to estimate the time required to reach steady state conditions in our enclosure setup. The 1,2-ISOPOOH deposition rate to the enclosure surface ($k_{dep,wall}$) becomes negligible after 91 min, corresponding to 20 residence times. In the case of MVK this occurs after 23 min (5 residence times). All experiments were performed in such a way that ample time was allowed in order to reach steady-state conditions before analyzing deposition rates of 1,2-ISOPOOH to plants. To minimize the effects of 1,2-ISOPOOH reemission from surfaces due to changes in humidity, only steady-state conditions were considered in each respective step for further analyses.

Photolytic loss of 1,2-ISOPOOH

We tested possible photolysis losses of 1,2-ISOPOOH (caused by irradiation) in the empty enclosure. For this purpose, we fumigated the empty enclosure with 1,2-ISOPOOH in darkness. Once the 1,2-ISOPOOH signals had stabilized after ~120 minutes, the light was switched on. We observed, however, no effect of the radiation on the 1,2-ISOPOOH volume mixing ratios in the enclosure.

Four-step protocol for the 1,2-ISOPOOH fumigation experiment

Each poplar plant was installed in the enclosure system shown in Supplementary Figure 1 several hours before starting an individual experiment, allowing the plant to adapt to the enclosure environment and recover from possible stress during the installation process. Before starting the fumigation run, we measured the "default" emissions of the gray poplars during darkness and illumination. We performed 1,2-ISOPOOH fumigation experiments following a four-step protocol for each plant (Supplementary Table 2). During step (A), 1,2-ISOPOOH from the liquid calibration unit (LCU) was analyzed directly with the SRI-ToF-MS *via* a bypass system, while the plant enclosure was fumigated with catalytically generated clean air (zero-air,). During steps (B), (C) and (D), the SRI-ToF-MS sampled air at the enclosure outlet. At step (B), a poplar plant was fumigated with 1,2-ISOPOOH, typically for 9-12 hours, under dark conditions (no light), followed by fumigation of the illuminated plant (step C) for another 9-12 hours. After performing steps (A), (B) and (C), the plant was removed from the enclosure in order to conduct background measurements of the empty enclosure (step D). An asterisk following the step label (e.g. A*) indicates that the air mixture was passed through a 1 m × ¼

inch (length × diameter) stainless steel tube kept at room temperature before being analyzed by the SRI-ToF-MS. As reported previously (*1*, *5*, *6*), under these conditions 1,2-ISOPOOH is converted efficiently to MVK and C_5-diols. For further analysis, we used the OVOC data averaged over 30-60 min at the end of each experimental step when OVOC signals had reached steady state conditions.

Representative poplar fumigation experiment

Supplementary Figure 3 depicts a typical fumigation cycle for a gray poplar plant, performed according to the four-step protocol. In step A, we enriched the air with 7.8±1.0 ppbv of 1,2-ISOPOOH across all replicates. The air flow leaving the LCU also contained 2.0±0.4 ppbv MVK and 0.2±0.1 ppbv C_5-diols as contaminants in the 1,2-ISOPOOH standard. Comparing the 1,2-ISOPOOH concentration in the outlet air of the fumigated empty enclosure (step D) with the corresponding 1,2-ISOPOOH values measured in the inlet air before the plant fumigation experiment (step A) revealed only minor (if any) losses to the enclosure system. On average only 6±5% of the input gaseous 1,2-ISOPOOH was lost to the enclosure surface when equilibration times of more than 2 hours were permitted. Upon starting the fumigation of the plant under dark conditions (step B), the mixing ratio of 1,2-ISOPOOH increased slowly over the course of several hours, reaching steady state after 8-10 hours. The mixing ratio of MVK reached steady state conditions (equal to the input concentration) after approximately 22 min, which is consistent with the time it takes for >99% air exchange of the enclosure ($5\tau \approx 22$ minutes). Instantaneously upon illumination the concentration of 1,2-ISOPOOH started to decrease. At the same time, the MVK signal showed a slight initial burst, followed by a decrease in emission, which leveled off to a ~1.5-fold increase from the original MVK concentration. MVK emissions were accompanied by a simultaneous increase in the MEK signal. When passing the air flow through metal tubing in steps A*, B*, C* and D*, the MVK and C_5-diol signal increased due to 1,2-ISOPOOH conversion on metal surfaces, while the MEK signal remained unchanged.

Calculation of emission dynamics

The deposition velocity v_d (cm s^{-1}) is commonly used to describe trace gas deposition to vegetation from the atmosphere (*7*), and is defined as the ratio between the flux Φ_i (representing the amount of compound i deposited to a unit surface area per unit time) and the local concentration c_i.

$$\Phi_i = -v_{\mathrm{d,i}} \cdot c_i \tag{3}$$

Similarly, as described in (*8*), for enclosure measurements the deposition velocity $v_{d,i}$ for compound i can be estimated from the flux Φ_i to the system (plant + enclosure surface) and the concentrations $c_{i,out}$ measured at the enclosure outlet:

$$v_{d,i} = \frac{-\Phi_i}{c_{i,out}}, [m\ s^{-1}] \tag{4}$$

Humidity corrected deposition fluxes Φ_i to the plant surfaces inside an enclosure are typically calculated from the difference in trace gas mixing ratios between the inlet ($c_{in,i}$) and outlet ($c_{out,i}$) in nmol mol^{-1}, taking into account the enclosed single-sided leaf area (LA, in m^2) of the plant,the gas flow (F, in mol s^{-1}) and w_e and w_o the mole fraction of water vapor entering and leaving the enclosure, respectively (*9*, *10*):

$$\Phi_i = \frac{F}{LA} \cdot \left(c_{in,i} - \frac{1 - w_e \cdot 10^{-3}}{1 - w_o \cdot 10^{-3}} c_{out,i} \right), [nmol\ m^{-2}\ s^{-1}] \quad (5)$$

To take into account possible losses to the enclosure surfaces, we adapted the commonly known formula for emission rates (Equation 5) to the following form (Equation 6) which follows the considerations of (*11*):

$$\Phi_i = \frac{F}{LA} \cdot \left(c_{out,i,BG} - \frac{1 - w_e \cdot 10^{-3}}{1 - w_o \cdot 10^{-3}} c_{out,i} \right), [nmol\ m^{-2} s^{-1}] \quad (6)$$

where $c_{out,i,BG}$ is the volume mixing ratio of substance i at the enclosure outlet during fumigation of the empty enclosure (step D, see below). Both background and plant experiments were performed under identical conditions in terms of 1,2-ISOPOOH mixing ratios and relative humidity in the inlet air. This background correction minimizes any potential errors caused by an underestimation of surface sinks.

Liquid Calibration Unit (LCU)

A liquid calibration unit (LCU, Ionicon Analytic GmbH, Innsbruck, Austria) allows quantitative evaporation of liquid standards into a gas stream to generate calibration mixtures. Here an LCU was used to evaporate an aqueous solution of 1,2-ISOPOOH into a synthetic air stream fed into the plant enclosure. The liquid flow of the 1,2-ISOPOOH solution was regulated and kept constant at 10 µL min^{-1}. Additionally, 40 µL min^{-1} of purified water were introduced into the synthetic air stream resulting in a relative humidity of approximately 35% at room temperature. The temperature in the evaporation chamber of the LCU was set to 50°C to avoid thermally-induced dissociation of 1,2-ISOPOOH.

Infrared gas analyzer (IRGA)

An infrared gas analyzer (LI-840A CO_2/H_2O Analyzer, LI-COR Inc., Lincoln NE, USA) sampled at 5 min intervals from either the inlet or outlet of the enclosure. CO_2 concentrations at the inlet were typically kept constant at 450 ppmv throughout the experiments. Typical relative humidities measured at the cuvette outlet ranged from 35-55 % in darkness to 50-60 % during the illuminated phase. Plants were illuminated by a true light lamp (Dakar, MT/HQI-T/D, Lanzini Illumazione, Brescia, Italy) to provide photosynthetically active radiation (PAR) of 400 µmol photons m^{-2} s^{-1} at the canopy level. Infrared light was shielded by a water filter to prevent radiative heating of the enclosure. PAR was measured with a sunshine sensor (Model SQ-326, Apogee Instruments, Logan, UT, USA).

Chemical ionization utilizing SRI-ToF-MS

Chemical ionization of 1,2-ISOPOOH with H_3O^+ and NO^+ leads to strong fragmentation. In contrast, using NH_4^+ as reagent ion, 1,2-ISOPOOH ($C_5H_{10}O_3$) undergoes an association reaction forming $NH_4^+(C_5H_{10}O_3)$ (m/z 136.097) product ions (*12*). 1,2-ISOPOOH decomposes at elevated temperatures (e.g., during GC analysis; (*1*)) or on stainless steel surfaces even at room temperature (*6*) to MVK and 3-methyl-1-butene-3,4-diol (C_5-diol, (*5*)). NH_4^+ reagent ions also undergo association reactions with these decomposition products of 1,2-ISOPOOH. MVK is detected as the $NH_4^+(C_4H_6O)$ (m/z 88.07) ion (*13*) and C_5-diols are found as $NH_4^+(C_5H_{10}O_2)$ (m/z 120.10) product ions. In the NH_4^+ reagent ion mode, the MVK and MACR isomers are

both detected as $NH_4^+(C_4H_6O)$ (m/z 88.07) product ions. In order to distinguish between MVK and MACR we used the NO^+ reagent ion mode, as the ketone MVK undergoes an association reaction forming $NO^+(C_4H_6O)$ (m/z 100.040) ions, while the aldehyde MACR performs hydride ion transfer forming $C_4H_5O^+$ (m/z 69.034) (*14*).
To distinguish MEK from isomers such as butanal and 2-methyl propanal, we used the reagent ion NO^+. Our instrument allows fast switching between different reagent ions. According to (*15*) the two isomers butanal and 2-methyl propanal undergo hydride abstraction forming the product ion $C_4H_7O^+$ (m/z 71.05). In contrast, MEK reacts with NO^+ via an association reaction forming C_4H_8O-NO^+ (m/z 102.05).

<u>Calibration of the NH_4^+-mode for SRI-ToF-MS</u>

We prepared a quantitative liquid solution of the pure 1,2-ISOPOOH standard with deionized water. The purity of the 1,2-ISOPOOH standard was verified by NMR spectroscopy in Innsbruck (working group Holger Kopacka, Institute of General, Inorganic and Theoretical Chemistry, University of Innsbruck) after shipment from the USA. The liquid solution was evaporated in the LCU into a defined synthetic air stream of 3 slpm. Additionally, we evaporated three different water amounts into the air stream resulting in absolute humidity in the range of 11 - 23 ppth. The sensitivity of 1,2-ISOPOOH at each humidity was then obtained from the slope of a linear fit through a scatter plot of the normalized product ion signals (ncps) *vs.* the 1,2-ISOPOOH volume mixing ratio. The sensitivity of the C_5-diol was obtained by passing a known amount of 1,2-ISOPOOH in air through stainless steel tubing (1 m length × ¼ inch diameter) kept at room temperature, leading to quantitative conversion of 1,2-ISOPOOH to MVK and C_5-diol. The sensitivity of MVK was obtained from dynamic dilution of a gas standard (Apel-Riemer Inc., Broomfield, USA) in humidified air. Errors were estimated by Gaussian propagation. An overview of the estimated sensitivities is given in Supplementary Table 3.

<u>Impurity determination for fumigation experiments</u>

For all fumigation experiments we used a 12 bottle bundle of synthetic air (5.0 grade). This synthetic air contains < 0.1 ppmv hydrocarbons (as CH_4) and the largest contamination is water vapor (< 5 ppmv). For each plant experiment we conducted a "blank" measurement of the empty enclosure. The SRI-TOF-MS limit of detection (LOD) for MVK and MEK was 0.03 ppbv, while the LOD for 1,2-ISOPOOH was 0.21 ppbv. Blank measurements of the empty enclosure with humidified synthetic air containing 480 ppm CO_2 revealed contaminants at m/z 88.07 (C_4H_6O-NH_4^+, MVK) and at m/z 90.09 (C_4H_8O-NH_4^+, MEK) of 0.09 ppbv and 0.07 ppbv, respectively. Contaminants at m/z 136.09 ($C_5H_{10}O_3$-NH_4^+, 1,2-ISOPOOH) were below the detection limit. During plant enclosure experiments observed ion signals are substantially higher than these back-ground signals.

Supplementary Methods - Construction of AOR Phylogenetic Trees and AOR Gene Expression Analysis

To detect AOR genes, sequence similarity searches (BLAST; e-value cutoff 1e-4) were conducted against the NCBI nr, dbEST and SRA databases using known amino acid sequences of genes encoding the chloroplast and cytosolic plant AOR proteins. Reciprocal BLAST searches were conducted to ensure that AOR represents the closest homologs to the identified bacterial and Bryophyta sequences in Angiosperms. To recover AOR sequences from *Quercus robur* and *Fagus sylvatica*, BLAST searches (e-value threshold 1e-30) were conducted against a local database containing whole genome and predicted protein sequences for these species

(*16*, *17*). Sequences for *Picea abies* and *Pinus taeda* were obtained from Congenie (http://congenie.org); *Salix purpurea* AOR sequences were obtained from Phytozome (https://phytozome.jgi.doe.gov). Multiple sequence alignments containing plant and bacterial (Supplementary Figure 10) and only plant (Figure 3) AOR genes were constructed using MUSCLE (*18*) and refined manually using Mesquite v. 3.51 (*19*). Maximum Likelihood (ML) AOR phylogenetic trees were reconstructed using RaxML (*20*) with bootstrapping (100 bootstrap replicates) and the LG amino acid substitution model. To discriminate between genes encoding cytosolic and plastid-targeted AOR, a targeting peptide prediction was conducted using TargetP 1.1 Server (*21*).
Expression information for the gray poplar chloroplasic and cytocolic AOR genes was derived from (*22*). Only samples collected in the light phase from plants maintained under normal control scenarios (AC: ambient CO_2; EC: enhanced CO_2) conditions were considered for analysis.

Supplementary Methods - Eddy Covariance VOC Flux Measurements

Calibration and data analysis of the PTR3

Isoprene and MEK sensitivities were calibrated as a function of humidity using a gas standard (Apel-Riemer Inc., Broomfield, USA), which was diluted in air with changing humidity. The humidity was varied from dry (synthetic air bottle) to tens of ppth during calibration covering the typical ambient humidity conditions. Water transport in eddies causes the sample humidity to correlate with vertical wind speed. This can induce artifacts in EC measurements if the analyzer shows humidity-dependent sensitivity. In order to accurately calibrate the PTR3-TOF VOC signals, a fast water-sensitive tracer (N_2H^+), which is produced in the PTR3-TOF, was regularly cross-calibrated against ambient humidity measurements with the IRGA (time resolution of seconds). This calibration of the PTR3-TOF signals was done at 10 Hz, resulting in a fast humidity trace. Data analysis was performed using multi-peak analysis routines (*23*), which only rely on single-ion counting within specified mass ranges and a subsequent correction of cross talk from neighboring mass peaks. This is important since typical mass spectra show fully developed peak shapes at the 10 Hz acquisition rate that can be peak-fitted. The resulting time-traces were calibrated as described above and used as input for "InnFlux", an eddy covariance flux routine developed by the group of Thomas Karl at the Faculty of Geo- and Atmospheric Sciences, University of Innsbruck, Austria.

Statistical analysis

Biological replication: for enclosure measurements with 1,2-ISOPOOH fumigation we used gas phase data for five biological replicates (for plant details see Supplementary Table 4). For AOR analysis we took samples from seven and five poplars (ISOPOOH and MVK fumigation, respectively). From each extract, three technical replicates were analyzed.

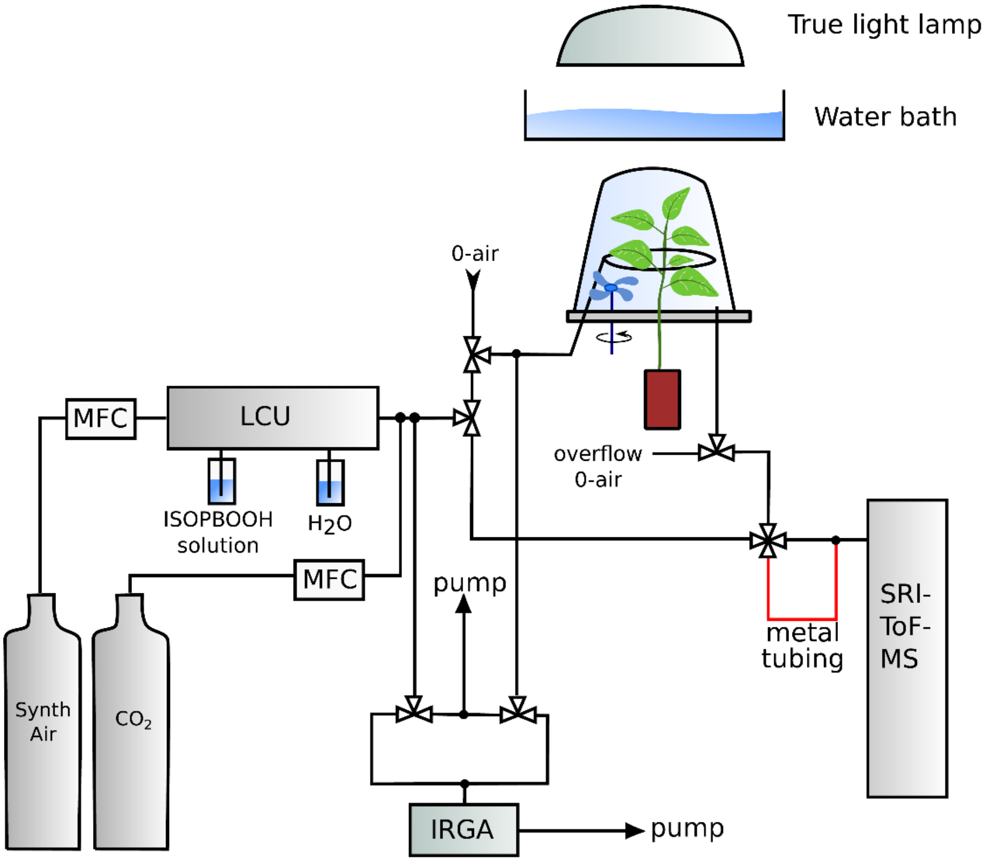

Supplementary Figure 1. Experimental design of enclosure measurements

A poplar plant was placed in the enclosure equipped with a Teflon fan for turbulent mixing. Gas flows of synthetic air and CO_2 were controlled by mass flow controllers (MFC). The liquid calibration unit (LCU) humidified the air stream (RH 35% at room temperature) and added ~7 ppbv 1,2-ISOPOOH. Switching valves directed the gas stream either before or after the enclosure to the SRI-TOF-MS with or without passing through a metal line. CO_2 and H_2O concentrations were analyzed with an infrared gas analyzer (IRGA) either before or after the enclosure. Plants were illuminated with a true light lamp. Infrared radiation was blocked by a water bath. The enclosure could be flushed with zero air (0-air).

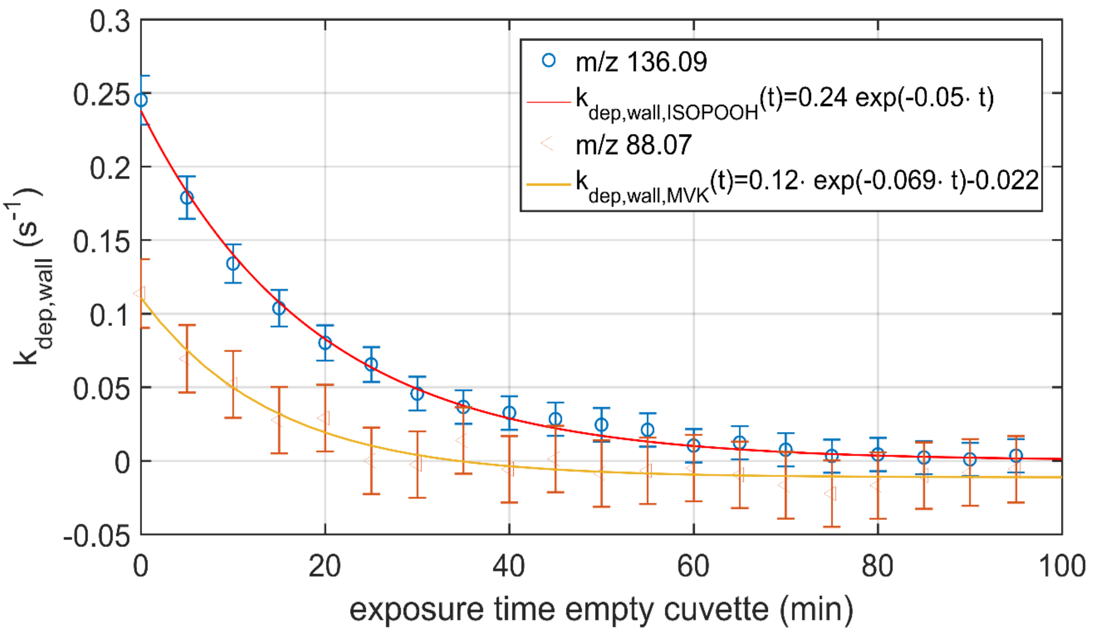

Supplementary Figure 2. Deposition rate to enclosure walls

Deposition rates $k_{dep,wall}$ for 1,2-ISOPOOH (m/z 136.09) and MVK (m/z 88.07) were obtained by fumigation of the empty enclosure with ~7 ppbv 1,2-ISOPOOH and with ~2 ppbv MVK. Error bars are calculated by Gaussian error propagation.

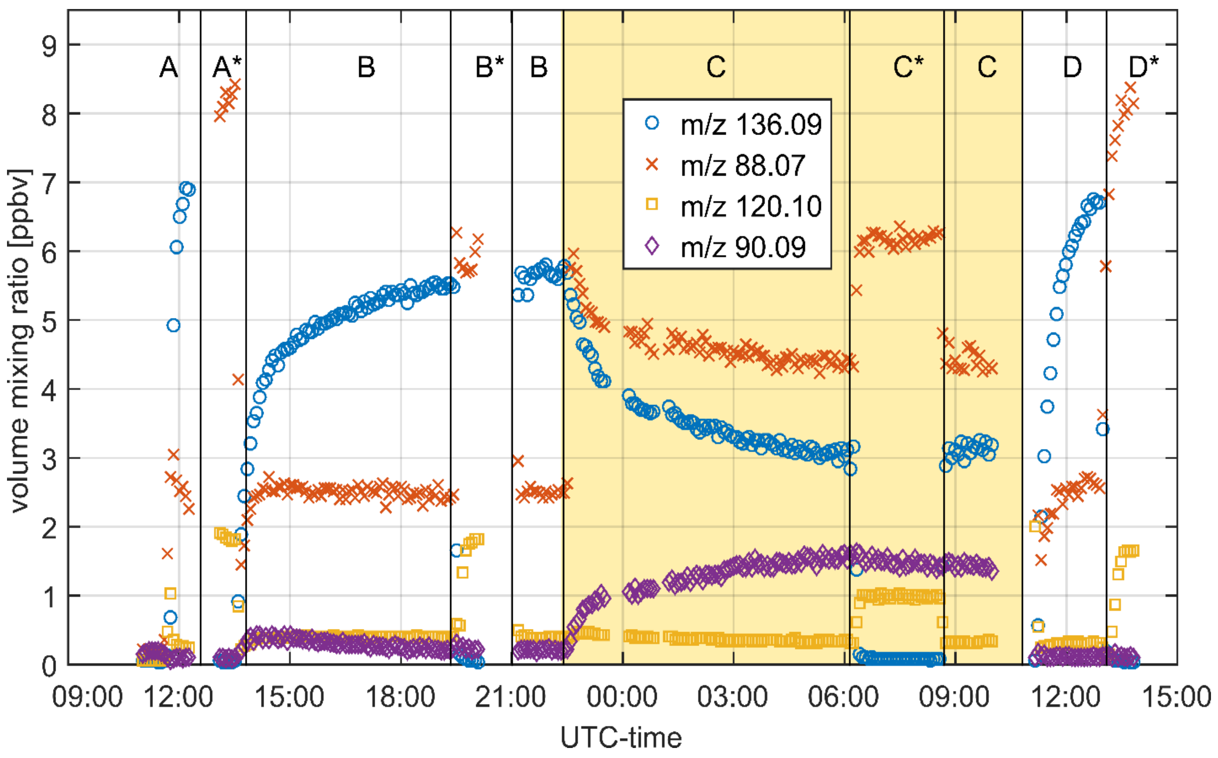

Supplementary Figure 3. Representative laboratory experimental run

Individual OVOC signals monitored during a representative poplar fumigation experiment. (A) Humidified air containing 7.1 ppbv 1,2-ISOPOOH (m/z 136.09) and 2.5 ppbv MVK (m/z 88.07) was directly analyzed prior to entering the enclosure. (A*) The sample flow was directed through a metal line (indicated by an asterisk) and 1,2-ISOPOOH was converted to MVK and C5 diols (m/z 120.10). During (B), the sample flow was introduced into the enclosure under dark conditions, with the air analyzed at the enclosure exit, showing a rather slow increase in 1,2-ISOPOOH. During B* the remaining 1,2-ISOPOOH (measured after the enclosure) was converted to MVK and C5 diols by passing the flow through the metal line. During (C), the light was switched on thus triggering stomatal opening (CO_2 was taken up; not shown), and 1,2-ISOPOOH decreased to 3 ppbv, while MVK (4.5 ppbv) and MEK (m/z 90.09) (~1.5 ppbv) increased. (D) Removing the plant and fumigating the empty enclosure in the dark led to 1,2-ISOPOOH and MVK signals as measured during (A).

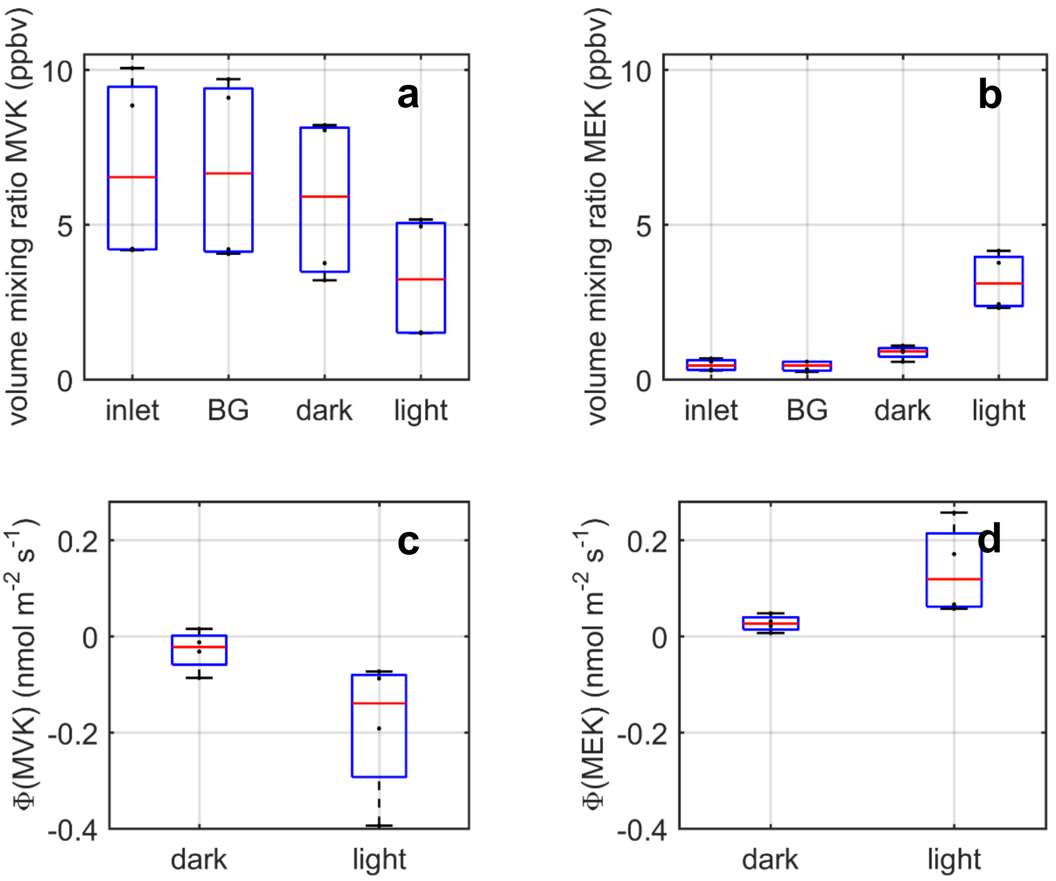

Supplementary Figure 4. Volume mixing ratios (VMR) and fluxes (Φ) of MVK and MEK in MVK fumigation experiments

Box plots of VMR (panels a,b) and deposition/emission fluxes (c,d) are shown for MVK and MEK during MVK fumigation. OVOCs were analyzed at the enclosure inlet and subsequently at the enclosure outlet during fumigation of the empty enclosure (BG) and the darkened/illuminated poplars. On each box, the red line indicates the median, bars show the minimum/maximum, and the blue box indicates the 25th and 75th percentiles of the sample data (N=5). The loss of MVK to the illuminated plant is statistically not significant (Tukey post hoc test). However, the Tukey post hoc test reveals a significant difference in MEK emissions between light and dark conditions (p=0.0001). Increases in both MEK and MVK fluxes under light conditions are statistically significant (p=0.0209 and p=0.0433, respectively).

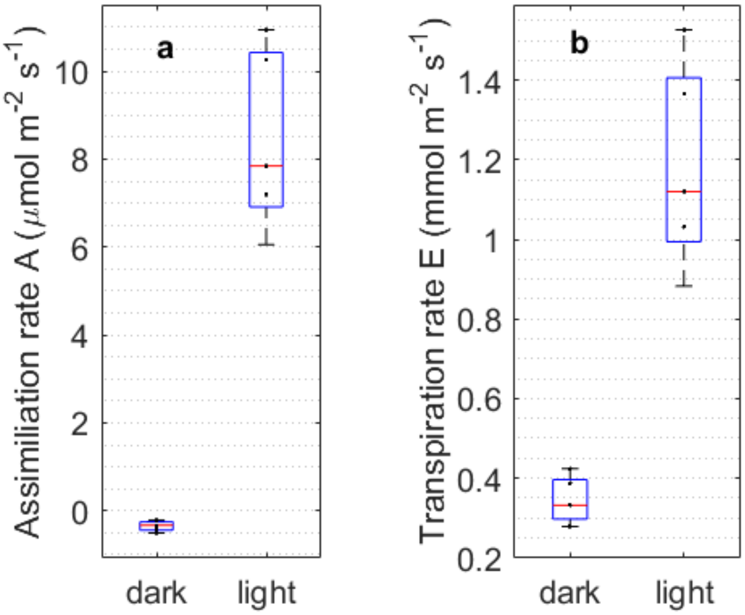

Supplementary Figure 5. Average net CO_2 assimilation and transpiration rates for 1,2-ISOPOOH fumigated gray poplars

The box plots show the CO_2 assimilation rate A (a) and transpirations rates E (b) among the fumigated gray poplars. Results are shown for both darkened and light conditions. On each box, the red line indicates the median, bars show the minimum/maximum, and the blue box indicates the 25th and 75th percentiles of the sample data (N=5).

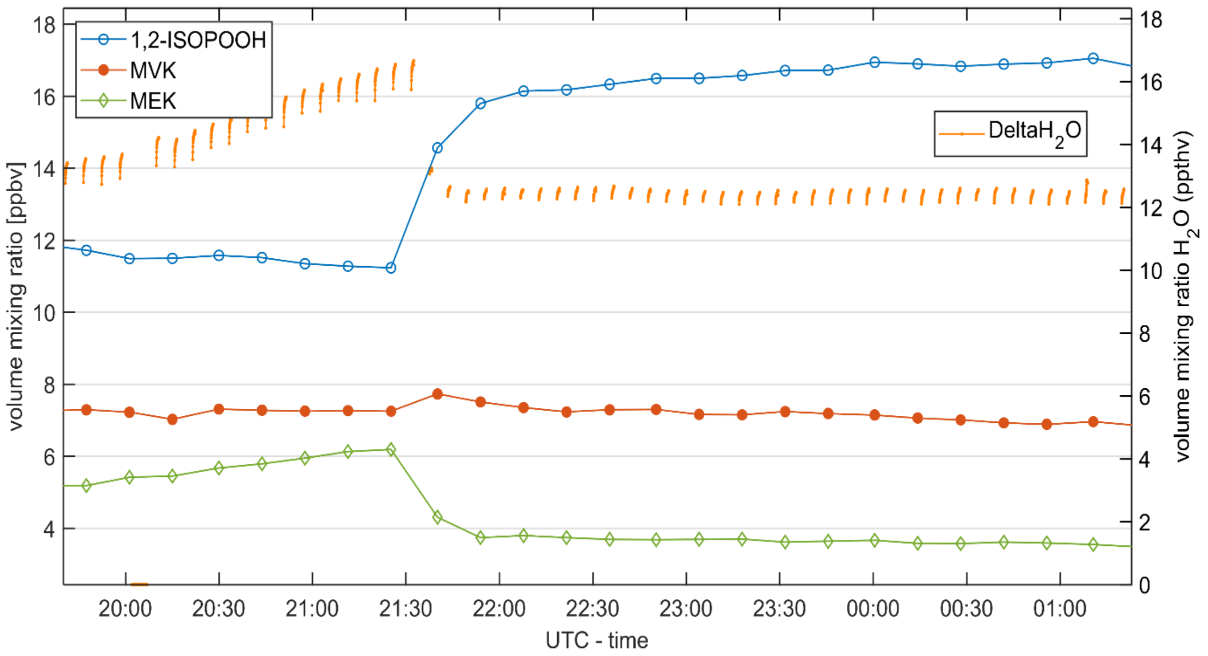

Supplementary Figure 6. Stomatal closing under light conditions during a poplar experiment.

Fumigation of a poplar plant with elevated 1,2-ISOPOOH under light conditions for several hours caused plant stress and stomatal closure. The resulting change in stomatal conductance is evident from the ΔH_2O (H_2O mixing ratio difference between inlet and outlet) increase. Stomatal closure was accompanied by an increase in 1,2-ISOPOOH, whereas MVK remained nearly unchanged and MEK levels decreased. These observations support our inference that 1,2-ISOPOOH is converted in the apoplast rather than on plant surfaces.

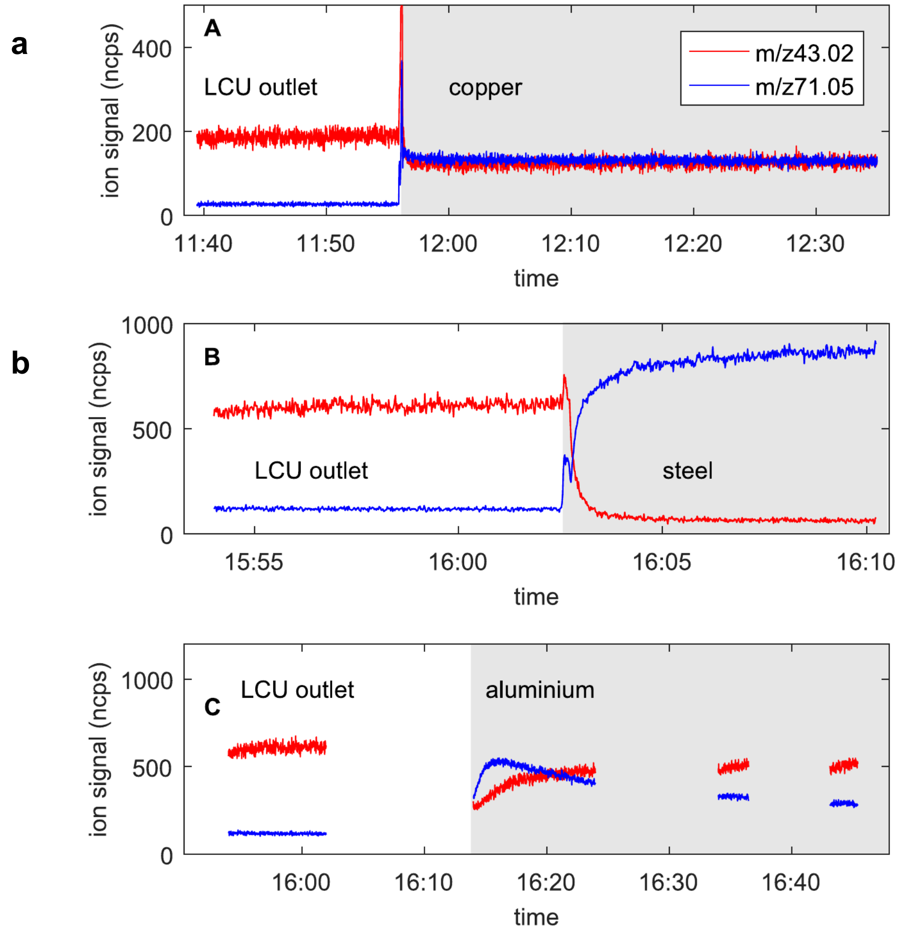

Supplementary Figure 7. 1,2-ISOPOOH reduction on different metals

1,2-ISOPOOH (m/z 43.02) conversion to MVK (m/z 71.05) on copper (panel a), steel (b) and aluminum (c). The cleaned metal cylinders were placed in the evaporation chamber of the LCU. 1,2-ISOPOOH was measured with the SRI-TOF-MS with H_3O^+ reagent ions and is detected as a fragment at m/z 43.02. Organic hydroperoxides are inherently unstable species and can undergo decomposition, such as homolytic cleavage of the weak peroxy (O-OH) bond. This reaction is catalyzed by metals. Copper and iron (both transition metals) are known to catalyze Fenton-type reactions of hydrogen peroxide (*24*). 1,2-ISOPOOH consistently undergoes conversion to MVK while in contact with steel or copper surfaces. Contact of 1,2-ISOPOOH with an aluminum surface results in an initial conversion to MVK followed by a recovery of the 1,2-ISOPOOH signal.

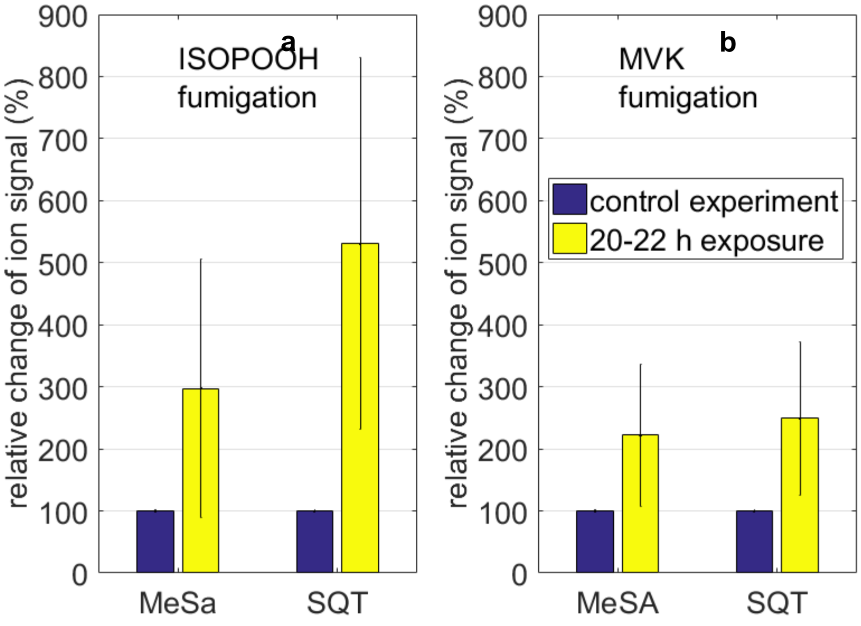

Supplementary Figure 8. MeSA and SQT signals following 1,2-ISOPOOH/MVK fumigation

Methyl salicylate (MeSA) and sesquiterpene (SQT) signals from 1,2-ISOPOOH (panel a, N=5 $\pm$ SD) and MVK (panel b, N=5 $\pm$ SD) fumigated gray poplars under control conditions and after 20-22h exposure (10-12 h dark conditions, 10-12 h illuminated conditions). As calibration gas standards were not available for MeSA and SQT we present data as relative changes in product ion signals.

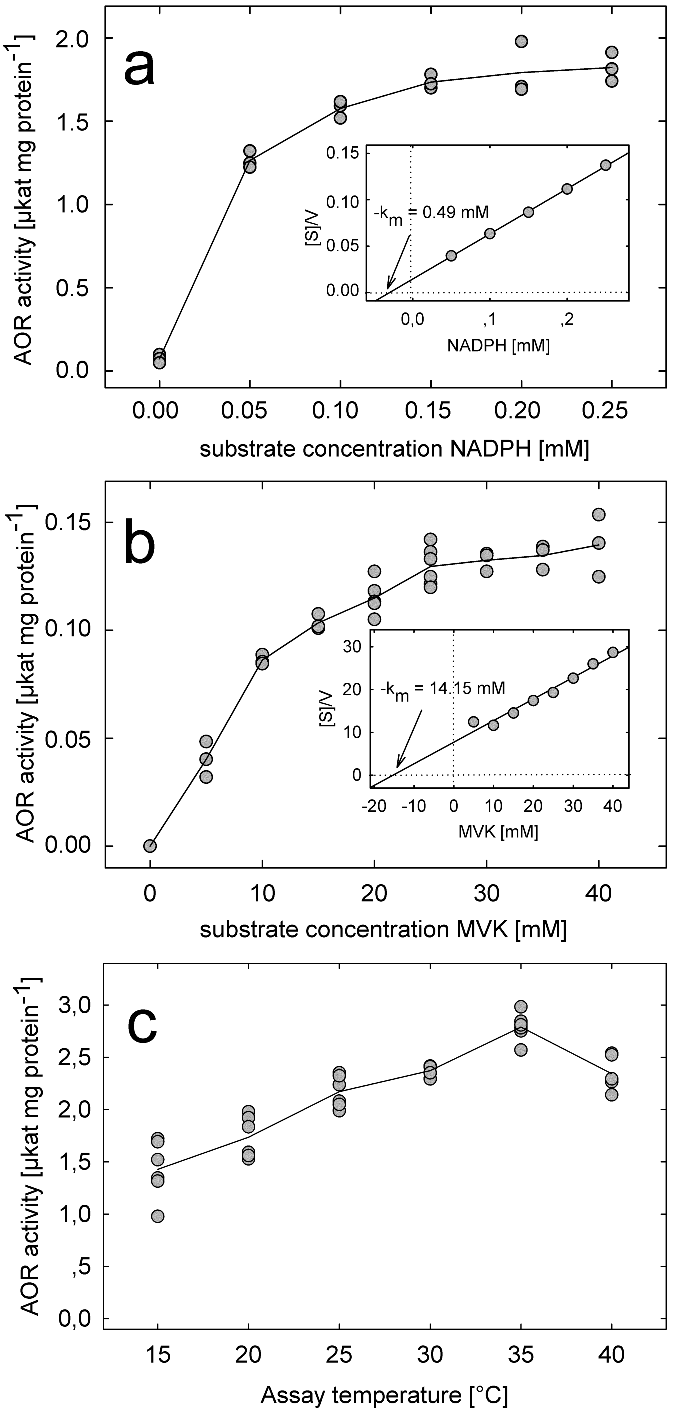

Supplementary Figure 9. Kinetic properties of the gray poplar AOR (EC 1.3.1.74, NADPH-dependent alkenal/one oxidoreductase) activity *in vitro*.

(a) Dependence of AOR activity on NADPH concentration. Insert: Hanes-Woolf diagram for determination of Michaelis constant (Km; indicates substrate concentration at half-maximal enzyme velocity). Km for NADPH 0.49 mM. (b) Dependence of AOR activity on methylvinylketone (MVK) concentration. Insert: Hanes-Woolf diagram with Km for MVK of 14.15 mM. (c) Temperature optimum of apparent *in vitro* AOR activity in poplar leaf extracts at saturating substrate concentrations. n = 3-6 replicates. The enzyme assays were performed according to (*25*).

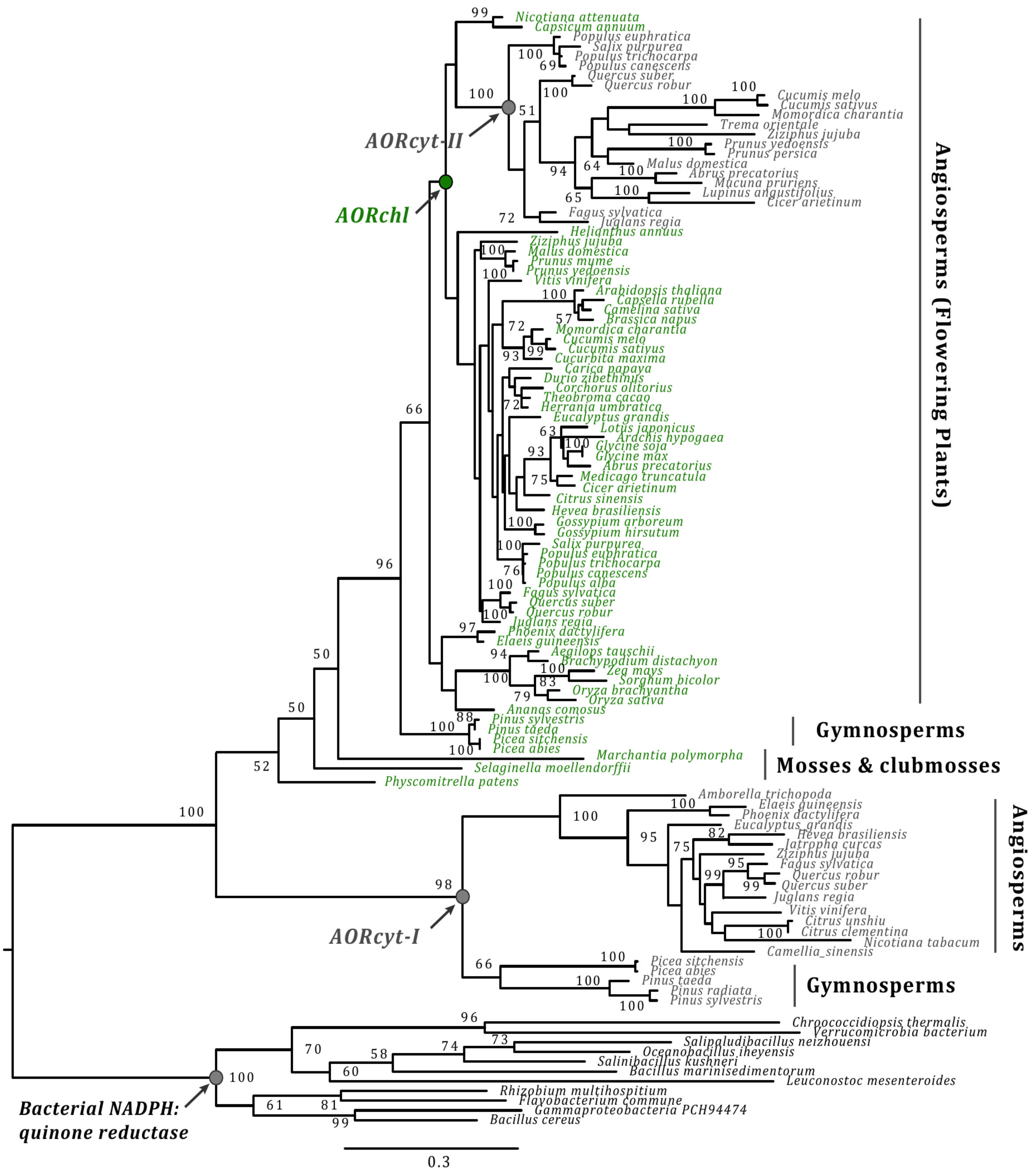

Supplementary Figure 10. AOR phylogenetic tree

Evolutionary history of genes encoding plastid and cytosolic AOR proteins. The AOR phylogeny was reconstructed using the maximum likelihood (ML) method and LG amino acid substitution model. The bacterial clade (black color) was used as outgroup. Gray coloring of the branch labels indicates genes encoding cytosolic AOR; green coloring indicates genes encoding choroplast AOR. Numbers at the tree branches indicate node bootstrap support. Basal nodes of the plant *AORchl*, *AORcyt-I* and *AORcyt-II* and bacterial NADPH: quinone reductase ortholog clusters are labeled with solid circles. Scale bar below the tree shows branch length. This phylogeny implies a bacterial origin of the plant AOR genes and several gene duplication events in *Embryophyte*. The first duplication event, which gave rise to the chloroplast and cytosolic AOR gene copies, is likely to have occurred during the early evolution of land plants. An additional cytosolic AOR copy has arisen *via* duplication of the chloroplast AOR copy in Angiosperms.

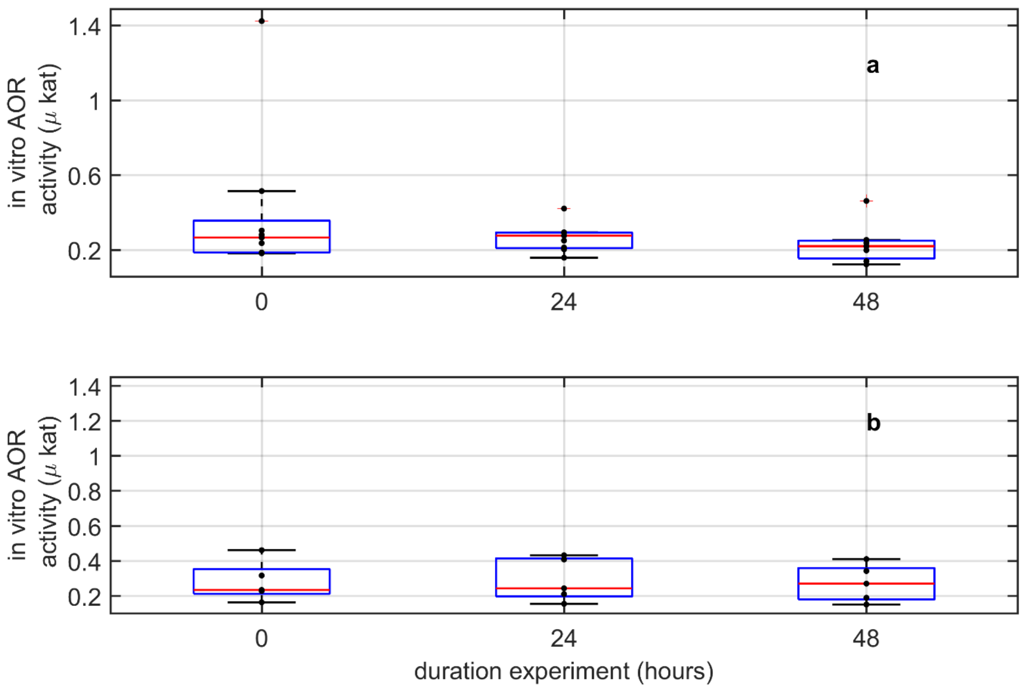

Supplementary Figure 11. *In vitro* AOR activity in fumigated gray poplar leaves

In vitro AOR activity in gray poplar leaf protein extracts. (a) Fumigation with 7.1 ppbv 1,2-ISOPOOH; (b) Fumigation with 2.5 ppbv MVK. Gray poplar leaves were sampled before starting fumigation (0 hours), after 24 hours of exposure and after 48 hours (24 hours of exposure followed by 24 hours of recovery). On each box, the red line indicates the median, bars show the minimum and maximum, the blue box indicates the 25th and 75th percentiles of the sample data, and red crosses indicate outliers (N= 7 (1,2-ISOPOOH); N=5 (MVK)).

a

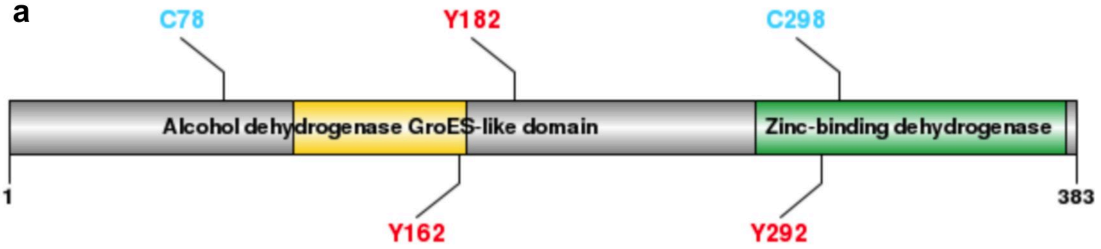

b

Site	Peptide	Score	Cutoff	PTM
78	IPSEMKACVYGEYGG	0.457	0.370	Cysteine S-Nitrosylation
162	LKEGDAVYGNINEKA	0.668	0.295	Tyrosine Nitration
182	QFGSLAEYTAVEEKL	0.452	0.295	Tyrosine Nitration
292	PEKFDVVYDAIGQCD	0.306	0.295	Tyrosine Nitration
78	VYDAIGQCDKAVKVV	0.411	0.370	Cysteine S-Nitrosylation

Supplementary Figure 12. Post-translational modification (PTM) of the *Populus x canescens* AORchl protein.

(**a**) Schematic representation of the *Populus x canescens AORchl* protein structure with predicted PTM sites. Numbers indicate positions of amino acids in the AORchl peptide sequence that can undergo PTM. (**b**) Predicted PTM site/ locus information. Prediction of the putative tyrosine- and tryptophan-nitration and S-nitrisylation sites was conducted using DeepNitro (*26*) at medium PTM prediction thresholds.

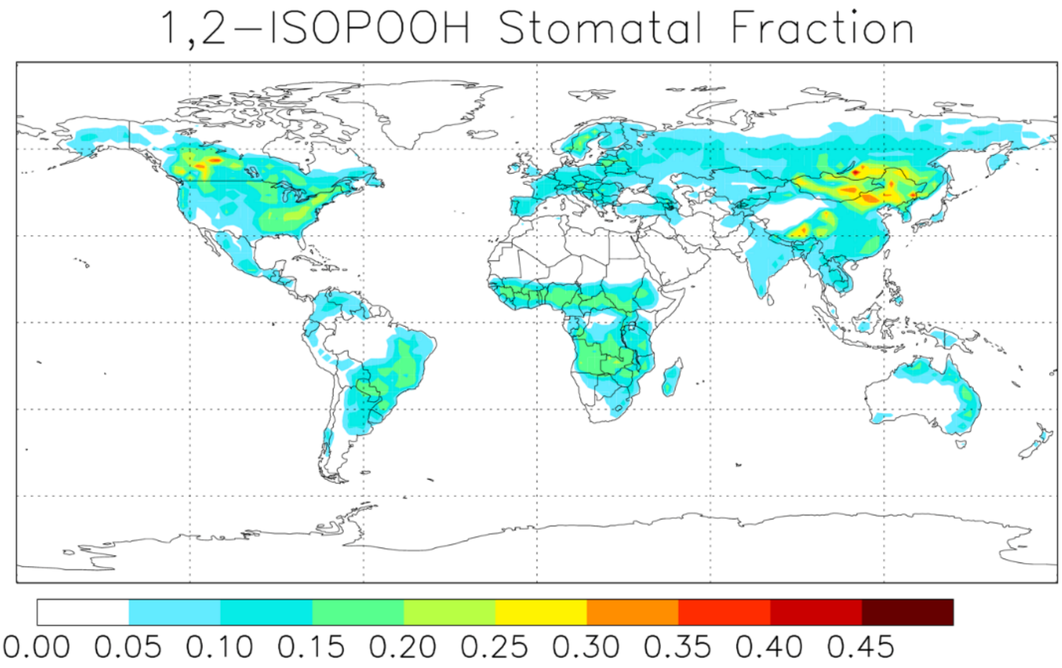

Supplementary Figure 13. Stomatal fraction of 1,2-ISOPOOH deposition

The stomatal fraction of dry deposited 1,2-ISOPOOH as simulated with the GEOS-Chem base-case run. By default, this approach uses a modified version of the resistance-based scheme from Wesely (*27*) to calculate dry deposition velocities. Values plotted reflect the stomatal fraction of deposition (i.e., stomatal conductance divided by total surface conductance) as a conductance-weighted mean across all land cover types within each model grid cell.

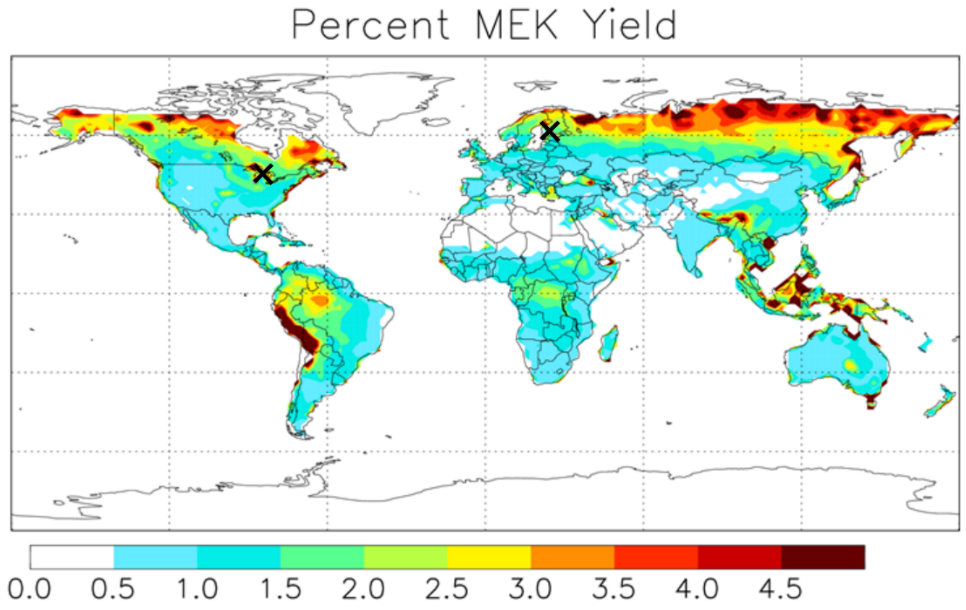

Supplementary Figure 14. MEK yield as simulated by GEOS-Chem

Global MEK yield expressed as a fraction of isoprene emissions as simulated by GEOS-Chem for 2017. Results shown use our experimentally obtained stomatal uptake values and assume that 100% of dry deposited MVK and 50% of dry deposited 1,2-ISOPOOH is enzymatically converted to MEK in terrestrial plants. Black crosses mark the two EC flux measurement sites (SMEAR II in Finland and PROPHET in US) discussed above. Elevated values are seen where OH is low and isoprene oxidation products undergo proportionately more deposition (e.g., high latitudes), and in low-emission locations subject to deposition from nearby high-emission areas (e.g., western South America).

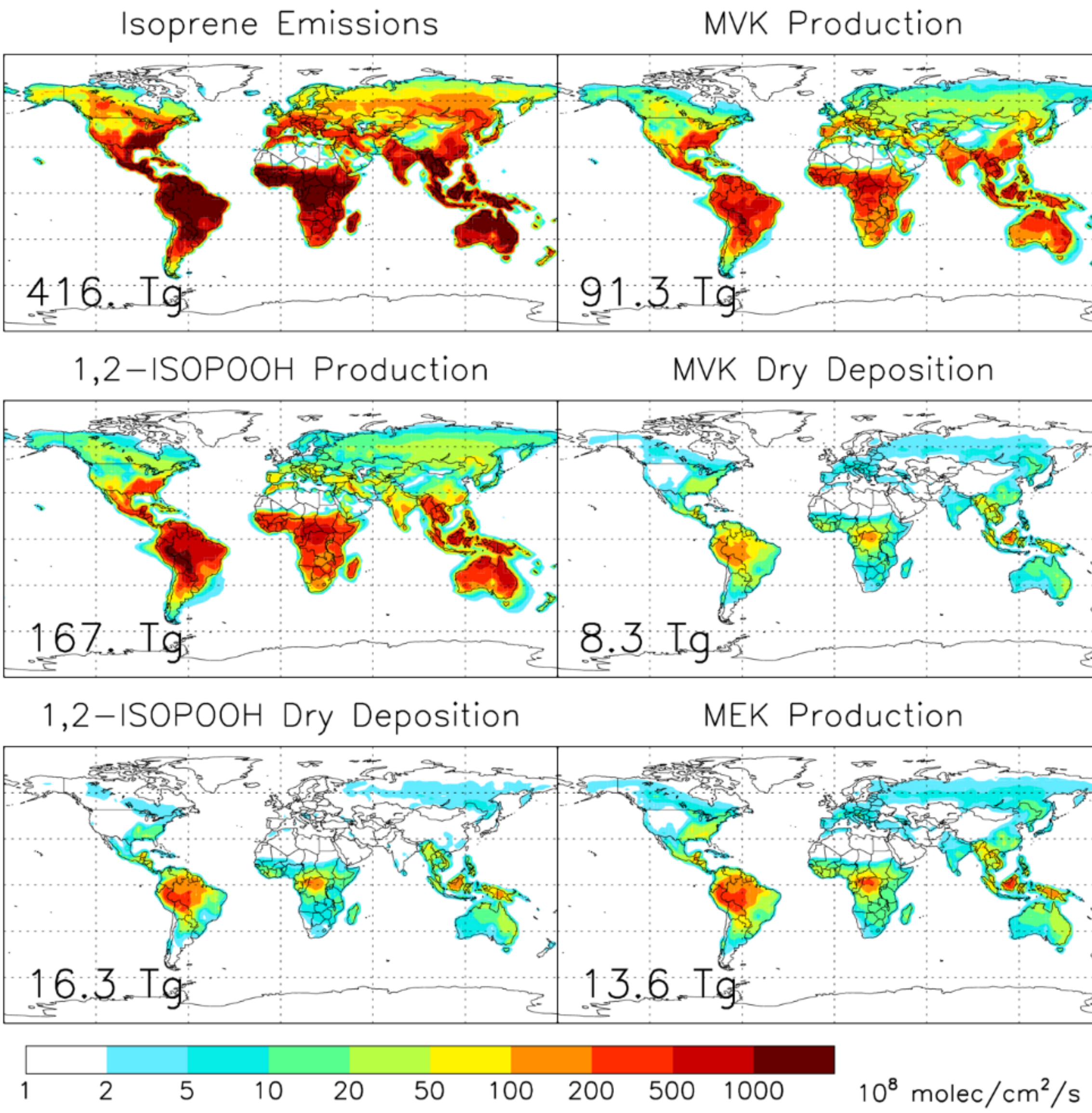

Supplementary Figure 15. GEOS-Chem run with default dry deposition treatment

GEOS-Chem base-case simulation for 2017 at 2º x 2.5º horizontal resolution. The model uses biogenic emissions from MEGANv2.1. Dry deposition is estimated using a modified version of the Wesely (*27*) scheme. MEK production in this simulation assumes that 100% of dry deposited MVK and 50% of dry deposited 1,2-ISOPOOH is subject to enzymatic conversion. Relevant parameters include H^* values of 1.7×10^6 M atm^{-1} for 1,2-ISOPOOH and 44 M atm^{-1} for MVK. Reactivity (f_0) values for both species are set to 1.0.

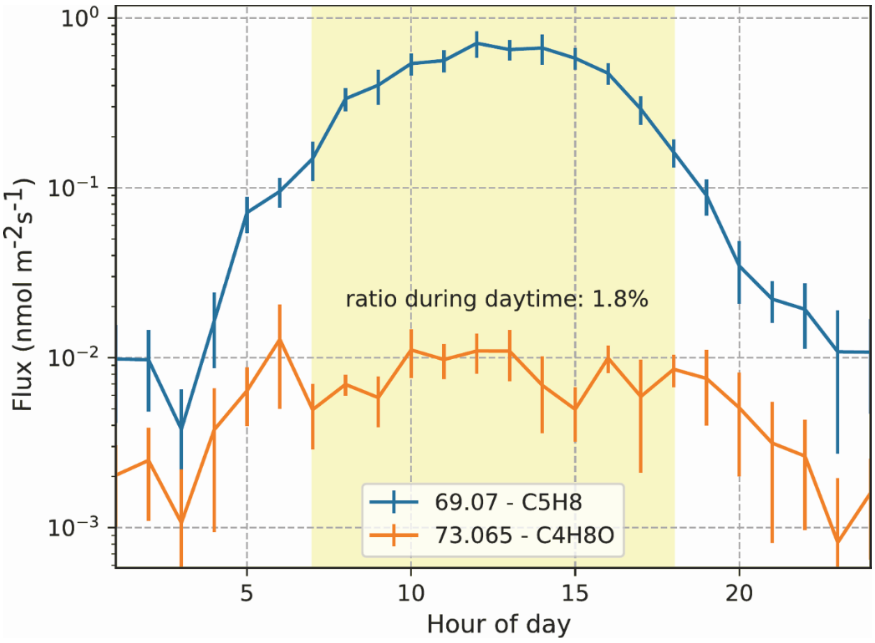

Supplementary Figure 16. EC flux measurements of isoprene and MEK at low isoprene emission site

Eddy covariance (EC) flux measurements of isoprene (m/z 69.07) and MEK (m/z 73.065) were performed at the SMEAR II station in Hyytiälä, Finland. During daytime (yellow) the MEK-to-isoprene diurnal flux ratio averages 1.8%. Data are averaged values (+/- SD) over five sunny days in May 2016.

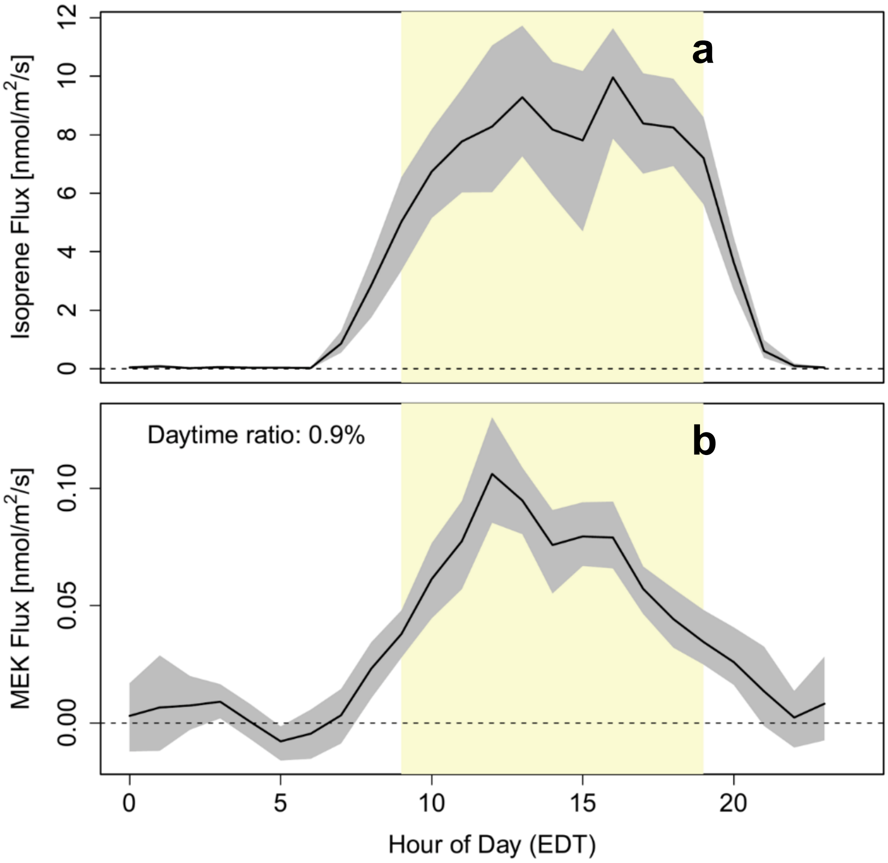

Supplementary Figure 17. EC flux measurements of isoprene and MEK at high isoprene emission site

Diurnal isoprene (panel a) and MEK (panel b) fluxes measured at the PROPHET site in Michigan, US in July 2016. The site is dominated by high isoprene-emitting species. The daytime (yellow shading) MEK-to-isoprene flux ratio averages 0.9%. Error ranges (gray) represent the 95% confidence interval. Data are mean values.

Supplementary Table 1. Wall deposition rates

Averaged wall deposition rates of ISOPOOH to the enclosure walls during fumigation of the empty enclosure across 5 experimental runs.

Exposure time (min)	$k_{dep,wall}$ (s^{-1})
0	0.23 ± 0.05
20	0.10 ± 0.03
40	0.05 ± 0.02
60	0.03 ± 0.01
120	0.02 ± 0.01

Supplementary Table 2. Four-step protocol of the ISOPOOH fumigation experiment

Steps performed during each experimental run of a gray poplar plant.

**Step**	**Description**
A	Analyzing air at the enclosure inlet
B	1,2-ISOPOOH fumigation of the shaded poplar, OVOCs sampled at the enclosure outlet
C	1,2-ISOPOOH fumigation of the illuminated poplar, OVOCs sampled at the enclosure outlet
D	1,2-ISOPOOH fumigation of the empty cuvette, OVOCs sampled at the enclosure outlet
*	Sampled air passes through a 1 m long stainless-steel tube before entering the SRI-ToF-MS

Supplementary Table 3. Sensitivities of the SRI-ToF-MS

Normalized sensitivities of MVK, MEK, acetone, 1,2-ISOPOOH and C_5-diol as a function of humidity for NH_4^+-mode measurements with the SRI-ToF-MS.

Humidity (ppth)		11	16.8	23.2
	LOD (ppbv)	ε (ncps/ppbv)	ε (ncps/ppbv)	ε (ncps/ppbv)
MVK	0.03 ± 0.01	8.5 ± 0.6	9.4 ± 0.6	10.62 ± 0.6
MEK	0.03 ± 0.01	11.8 ± 0.2	12.8 ± 0.6	14.06 ± 0.5
Acetone	0.04 ± 0.02	6.6 ± 0.3	7.65 ± 0.7	9.054 ± 0.7
1,2-ISOPOOH	0.21 ± 0.05	25.6 ± 6.8	21.8 ± 5.9	20.26 ± 5.4
C_5-diol		21.2 ± 9.3	20 ± 6.9	13.68 ± 7.5

Supplementary Table 4. Plant details

Leaf areas for the analyzed poplars were calculated by the free tool LeafArea Calculator (https://sites.google.com/site/ptrtof/file-cabinet) which is described in (*3*).

Poplar (No.)	Leaf area (cm^2)	Fumigation gas	AOR analysis
1	242	1,2-ISOPOOH	-
2	188	1,2-ISOPOOH	-
3	215	1,2-ISOPOOH	-
4	350	1,2-ISOPOOH	-
5	242	1,2-ISOPOOH	-
6	768	1,2-ISOPOOH	X
7	2101	1,2-ISOPOOH	X
8	1983	1,2-ISOPOOH	X
9	1849	1,2-ISOPOOH	X
10	1731	1,2-ISOPOOH	X
11	1629	1,2-ISOPOOH	X
12	1121	1,2-ISOPOOH	X
13	1232	MVK	X
14	968	MVK	X
15	416	MVK	X
16	665	MVK	X
17	1121	MVK	X

Supplementary Table 5. Sequence accession and BVOC emission information

Genes encoding chloroplastic (AORchl) and cytosolic AOR (AORcyt-I and AORcyt-II) proteins in plants emitting different BVOCs.

Species	Data-base	Accession nr / genomic coordinates	Gene description	Plastid-targeting signal (TP)	VOC emission information	
					VOC	Ref
Arabidopsis thaliana	NCBI nr	OAP18379.1	AOR	TP		
Elaeis guineensis	NCBI nr	XP_029123251.1	2-methylene-furan-3-one reductase	noTP	isoprene	(*28*)
Elaeis guineensis	NCBI nr	XP_010925514.1	2-methylene-furan-3-one reductase	TP	isoprene	(*28*)
Eucalyptus grandis	NCBI nr	Eucgr.I01800.1.p	2-methylene-furan-3-one reductase	noTP	isoprene, monoterpenes	(*29*)
Eucalyptus grandis	NCBI nr	Eucgr.I01801.1.p	2-methylene-furan-3-one reductase	noTP	isoprene, monoterpenes	(*29*)
Eucalyptus grandis	NCBI nr	Eucgr.E01364.1.p	2-methylene-furan-3-one reductase isoform X1	noTP	isoprene, monoterpenes	(*29*)
Eucalyptus grandis	NCBI nr	XP_010023565.1	PREDICTED: 2-methylene-furan-3-one reductase	TP	isoprene, monoterpenes	(*29*)
Fagus sylvatica	Beech genome*	FSB010890801	2-methylene-furan-3-one reductase-like|chloroplast stroma;thylakoid	noTP	monoterpenes	(*29*)
Fagus sylvatica	Beech genome*	FSB010890501	2-methylene-furan-3-one reductase-like|NADPH:quinone reductase activity	noTP	monoterpenes	(*29*)
Fagus sylvatica	Beech genome*	FSB016186801	2-methylene-furan-3-one reductase-like	noTP	monoterpenes	(*29*)
Fagus sylvatica	Beech genome*	FSB015711501	2-methylene-furan-3-one reductase-like|chloroplast stroma;thylakoid	TP	monoterpenes	(*29*)
Helianthus annuus	NCBI nr	XP_022011957.1	2-methylene-furan-3-one reductase-like	TP	isoprene, monoterpenes	(*30*)
Hevea brasiliensis	NCBI nr	XP_021668587.1	2-methylene-furan-3-one reductase-like	noTP	monoterpenes	(*31*)
Hevea brasiliensis	NCBI nr	XP_021645268.1	2-methylene-furan-3-one reductase-like	TP	monoterpenes	(*31*)
Oryza brachyantha	NCBI nr	XP_006659368.1	2-methylene-furan-3-one reductase	TP		
	NCBI nr	XP_015650762.1	2-methylene-furan-3-one reductase	TP		
Phoenix dactylifera	NCBI nr	XP_008803292.1	2-methylene-furan-3-one reductase-like isoform X1	noTP	isoprene	(*32*)
Phoenix_dactylifera	NCBI nr	XP_008801828.1	1-methylene-furan-3-one reductase	TP	isoprene	(*32*)
Picea abies	Congenie	MA_10433694g0010	NA	noTP	isoprene, monoterpenes	(*29*)
Picea abies	Congenie	MA_207070g0010	NA	noTP	isoprene, monoterpenes	(*29*)

Picea sitchensis	NCBI nr	ABK24706.1	unknown	noTP	isoprene, monoterpenes	(*29*)
Picea sitchensis	NCBI dbEST	DR524779.1	NA	noTP	isoprene, monoterpenes	(*29*)
Pinus radiata	NCBI dbEST	FE520506.1	NA	noTP	monoterpenes	(*29*)
Pinus sylvestris	NCBI dbEST	HE631563, HE627609	NA	noTP	monoterpenes	(*29*) (*33*)
Pinus sylvestris	NCBI dbEST	HE634642,HE629950	NA	noTP	monoterpenes	(*29*) (*33*)
Pinus taeda	Congenie	PITA_000008068	NA	noTP	monoterpenes	(*34*)
Pinus taeda	Congenie	PITA_000018354	NA	noTP	monoterpenes	(*34*)
Pinus taeda	Congenie	PITA_000040712	NA	noTP	monoterpenes	(*34*)
Populus alba	NCBI nr	TKR83752.1	hypothetical protein D5086_0000265230	noTP	isoprene	(*33*)
Populus alba	NCBI nr	TKS15680.1	hypothetical protein	TP	isoprene	(*33*)
Populus canescens	NCBI SRA	SRX5822327-SRX5822330	NA	noTP	isoprene	(*33*)
Populus canescens	NCBI SRA	SRX5820660-SRX5820707	NA	TP	isoprene	(*33*)
Populus euphratica	NCBI nr	XP_011002607.1	PREDICTED: 2-methylene-furan-3-one reductase-like	noTP	monoterpenes	(*35*)
Populus euphratica	NCBI nr	XP_011029885.1	PREDICTED: 2-methylene-furan-3-one reductase-like	noTP	monoterpenes	(*35*)
Populus trichocarpa	NCBI nr	XP_006379865.2	2-methylene-furan-3-one reductase	noTP	isoprene	(*29*)
Populus trichocarpa	NCBI nr	XP_002323668.2	2-methylene-furan-3-one reductase	TP	isoprene	(*29*)
Quercus robur	Oak genome**	Qrob_P0187340.2270	NA	noTP	isoprene	(*29*)
Quercus robur	Oak genome**	Qrob_P0415420.2285	NA	noTP	isoprene	(*29*)
Quercus robur	Oak genome**	Qrob_Chr03:49527575-49527850	NA	TP	isoprene	(*29*)
Quercus suber	NCBI nr	XP_023895222.1	2-methylene-furan-3-one reductase-like	noTP	monoterpenes	(*36*)
Quercus suber	NCBI nr	XP_023897000.1	2-methylene-furan-3-one reductase-like	noTP	monoterpenes	(*36*)
Quercus suber	NCBI nr	XP_023917302.1	2-methylene-furan-3-one reductase-like	TP	monoterpenes	(*36*)
Salix purpurea	Phyto-zome	SapurV1A.0580s0100.1.p	quinone oxidoreductase-like protein	noTP	isoprene	(*33*)
Salix purpurea	Phyto-zome	SapurV1A.0065s0430.1.p	alcohol dehydro-genase and quinone reductase-like medium chain degydrogenase family/reductase	TP	isoprene	(*33*)
Zea mays	NCBI nr	NP_001151204.1	quinone oxidoreductase-like protein At1g23740	TP		

* *Fagus sylvatica* (European Beech) genome resource (http://www.beechgenome.net/; (*17*))
** Oak genome sequencing (http://www.oakgenome.fr/; (*16*))

Supplementary references

1. J. C. Rivera-Rios, T. B. Nguyen, J. D. Crounse, W. Jud, *et al.*, Conversion of hydroperoxides to carbonyls in field and laboratory instrumentation: Observational bias in diagnosing pristine versus anthropogenically controlled atmospheric chemistry. *Geophys. Res. Lett.* **41**, 8645–8651 (2014).
2. B. Bonn, S. Sun, W. Haunold, R. Sitals, *et al.*, COMPASS - COMparative Particle formation in the Atmosphere using portable Simulation chamber Study techniques. *Atmos. Meas. Tech.* **6**, 3407–3423 (2013).
3. W. Jud, J. B. Winkler, B. Niederbacher, S. Niederbacher, *et al.*, Volatilomics : a non - invasive technique for screening plant phenotypic traits. *Plant Methods*, 1–18 (2018).
4. M. Graus, thesis, University of Innsbruck (2005).
5. J. M. St. Clair, J. C. Rivera-Rios, J. D. Crounse, H. C. Knap, *et al.*, Kinetics and products of the reaction of the first-generation isoprene hydroxy hydroperoxide (ISOPOOH) with OH. *J. Phys. Chem. A*. **120**, 1441–1451 (2016).
6. A. K. Bernhammer, M. Breitenlechner, F. N. Keutsch, A. Hansel, Technical note: Conversion of isoprene hydroxy hydroperoxides (ISOPOOHs) on metal environmental simulation chamber walls. *Atmos. Chem. Phys.* **17**, 4053–4062 (2017).
7. J. H. Seinfeld, S. N. Pandis, *Atmospheric chemistry and physics : from air pollution to climate change* (John Wiley & Sons, 2012).
8. A. Rondón, C. Johansson, L. Granat, Dry deposition of nitrogen dioxide and ozone to coniferous forests. *J. Geophys. Res.* **98**, 5159–5172 (1993).
9. S. Von Caemmerer, G. D. Farquhar, Some relationships between the biochemistry of photosynthesis and the gas exchange of leaves. *Planta*. **153**, 376–387 (1981).
10. J. T. Ball, in *Stomatal function*, E. Zeiger, G. D. Farquhar, I. R. Cowan, Eds. (Stanford University Press Stanford, California, 1987), pp. 445–476.
11. S. Sun, A. Moravek, L. von der Heyden, A. Held, *et al.*, Twin-cuvette measurement technique for investigation of dry deposition of O_3 and PAN to plant leaves under controlled humidity conditions. *Atmos. Meas. Tech.* **9**, 599–617 (2016).
12. S. Zhou, J. C. Rivera-Rios, F. N. Keutsch, J. P. D. Abbatt, Identification of organic hydroperoxides and peroxy acids using atmospheric pressure chemical ionization–tandem mass spectrometry (APCI-MS/MS): application to secondary organic aerosol. *Atmos. Meas. Tech.* **11**, 3081–3089 (2018).
13. E. Canaval, N. Hyttinen, B. Schmidbauer, L. Fischer, *et al.*, NH_4^+ association and proton transfer reactions with a series of oganic molecules. *Front. Chem.* **7**, 191 (2019).
14. W. Jud, L. Fischer, E. Canaval, G. Wohlfahrt, *et al.*, Plant surface reactions: An opportunistic ozone defence mechanism impacting atmospheric chemistry. *Atmos. Chem. Phys.* **16**, 277–292 (2016).
15. P. Španěl, J. M. V. Doren, D. Smith, A selected ion flow tube study of the reactions of H_3O^+, NO^+, and O_2^+ with saturated and unsaturated aldehydes and subsequent hydration of the product ions. *Int. J. Mass Spectrom.* **213**, 163–176 (2002).
16. C. Plomion, J.-M. Aury, J. Amselem, T. Leroy, *et al.*, Oak genome reveals facets of long lifespan. *Nat. Plants*. **4**, 440–452 (2018).
17. B. Mishra, D. K. Gupta, M. Pfenninger, T. Hickler, *et al.*, A reference genome of the European beech (*Fagus sylvatica L.*). *Gigascience*. **7** (2018), pp. 1–8.
18. R. C. Edgar, MUSCLE: multiple sequence alignment with high accuracy and high throughput. *Nucleic Acids Res.* **32**, 1792–1797 (2004).
19. W. P. Maddison, D. R. Maddison, Mesquite: a modular system for evolutionary analysis. Version 3.51 (2018), (available at http://www.mesquiteproject.org).
20. A. Stamatakis, RAxML version 8: a tool for phylogenetic analysis and post-analysis of

large phylogenies. *Bioinformatics*. **30**, 1312–1313 (2014).
21. O. Emanuelsson, S. Brunak, G. von Heijne, H. Nielsen, Locating proteins in the cell using TargetP, SignalP and related tools. *Nat. Protoc.* **2**, 953–971 (2007).
22. E. Georgii, K. Kugler, M. Pfeifer, E. Vanzo, *et al.*, The systems architecture of molecular memory in poplar after abiotic stress. *Plant Cell*. **31**, 346–367 (2019).
23. D. Stolzenburg, L. Fischer, A. L. Vogel, M. Heinritzi, *et al.*, Rapid growth of organic aerosol nanoparticles over a wide tropospheric temperature range. *Proc. Natl. Acad. Sci.* **115**, 9122–9127 (2018).
24. A. N. Pham, G. Xing, C. J. Miller, T. D. Waite, Fenton-like copper redox chemistry revisited: Hydrogen peroxide and superoxide mediation of copper-catalyzed oxidant production. *J. Catal.* **301**, 54–64 (2013).
25. Y. Yamauchi, A. Hasegawa, A. Taninaka, M. Mizutani, *et al.*, NADPH-dependent reductases involved in the detoxification of reactive carbonyls in plants. *J. Biol. Chem.* **286**, 6999–7009 (2011).
26. Y. Xie, X. Luo, Y. Li, L. Chen, *et al.*, DeepNitro: Prediction of Protein Nitration and Nitrosylation Sites by Deep Learning. *Genomics. Proteomics Bioinformatics*. **16**, 294–306 (2018).
27. M. L. Wesely, Parameterization of surface resistances to gaseous dry deposition in regional-scale numerical models. *Atmos. Environ.* **23**, 1293–1304 (1989).
28. M. J. Wilkinson, S. M. Owen, M. Possell, J. Hartwell, *et al.*, Circadian control of isoprene emissions from oil palm (Elaeis guineensis). *Plant J.* **47**, 960–968 (2006).
29. R. K. Monson, R. T. Jones, T. N. Rosenstiel, J. P. Schnitzler, Why only some plants emit isoprene. *Plant, Cell Environ.* **36**, 503–516 (2013).
30. G. Schuh, A. C. Heiden, T. Hoffmann, J. Kahl, *et al.*, Emissions of volatile organic compounds from sunflower and beech: Dependence on temperature and light intensity. *J. Atmos. Chem.* **27**, 291–318 (1997).
31. Y. F. Wang, S. M. Owen, Q. J. Li, J. Peñuelas, Monoterpene emissions from rubber trees (Hevea brasiliensis) in a changing landscape and climate: Chemical speciation and environmental control. *Glob. Chang. Biol.* **13**, 2270–2282 (2007).
32. L. Arab, J. Kreuzwieser, J. Kruse, I. Zimmer, *et al.*, Acclimation to heat and drought—Lessons to learn from the date palm (*Phoenix dactylifera*). *Environ. Exp. Bot.* **125**, 20–30 (2016).
33. F. Loreto, F. Bagnoli, C. Calfapietra, D. Cafasso, *et al.*, Isoprenoid emission in hygrophyte and xerophyte European woody flora: ecological and evolutionary implications. *Glob. Ecol. Biogeogr.* **23**, 334–345 (2014).
34. K. G. S. Dani, I. M. Jamie, I. C. Prentice, B. J. Atwell, Evolution of isoprene emission capacity in plants. *Trends Plant Sci.* **19**, 439–446 (2014).
35. K. Behnke, A. Ghirardo, D. Janz, B. Kanawati, *et al.*, Isoprene function in two contrasting poplars under salt and sunflecks. *Tree Physiol.* **33**, 562–578 (2013).
36. S. Welter, A. Bracho-Nunez, C. Mir, I. Zimmer, *et al.*, The diversification of terpene emissions in Mediterranean oaks: lessons from a study of *Quercus suber*, *Quercus canariensis* and its hybrid *Quercus afares*. *Tree Physiol.* **32**, 1082–1091 (2012).
